# Intrapartum antibiotic prophylaxis to prevent Group B streptococcal infections in newborn infants: a systematic review and meta-analysis comparing various strategies

**DOI:** 10.1016/j.eclinm.2024.102748

**Published:** 2024-07-28

**Authors:** Timothy J.R. Panneflek, Gea F. Hasperhoven, Yamikani Chimwaza, Connor Allen, Tina Lavin, Arjan B. te Pas, Vincent Bekker, Thomas van den Akker

**Affiliations:** a Division of Neonatology, Department of Paediatrics, Willem-Alexander Children's Hospital, Leiden University Medical Centre, Leiden, the Netherlands; b Department of Neonatal and Paediatric Intensive Care, Erasmus Medical Centre – Sophia Children's Hospital, Rotterdam, the Netherlands; c Malawi Liverpool Wellcome Clinical Research Programme, Blantyre, Malawi; d The Department of Medicine, Nursing and Health Sciences, Monash University, Melbourne, Australia; e UNDP/UNFPA/UNICEF/WHO/World Bank Special Programme of Research, Development and Research Training in Human Reproduction (HRP), Department of Sexual and Reproductive Health and Research, World Health Organisation, Geneva, Switzerland; f Department of Obstetrics, Leiden University Medical Centre, Leiden, the Netherlands; g Athena Institute, VU University, Amsterdam, the Netherlands

**Keywords:** Group B streptococcus, Early-onset neonatal sepsis, Antibiotic prophylaxis, Newborn infant, Risk-based, Screening

## Abstract

**Background:**

Early-onset Group B Streptococcus (EOGBS) infection leads to substantial morbidity and mortality in newborn infants. Intrapartum antibiotic prophylaxis (IAP) prevents EOGBS infection, but IAP strategies vary. The approach to the provision of IAP can be risk-based, universal or a combination of the two strategies. Previous systematic reviews reported that universal strategies might be most optimal in lowering EOGBS infection, but there is no consensus. Therefore, we aimed to provide up-to-date evidence on effectiveness of different strategies by comparing perinatal outcomes.

**Methods:**

A systematic search for EOGBS prevention strategies was performed in MEDLINE, Embase and Web of Science on May 2024. Studies were included if they reported on different strategies and outcomes of interest, including EOGBS infection, IAP administration and antimicrobial resistance regardless of publication date. Summary data was extracted from published reports. Study quality was assessed using the ROBINS-I tool. Random-effects meta-analyses were used to determine risk ratios (RR) and 95%-confidence intervals. PROSPERO registration CRD42023411806.

**Findings:**

A total of 6293 records were identified, of which 72 observational studies were included for synthesis with more than 10 million live births. Meta-analysis demonstrated that implementation of any strategy (n = 34 studies, RR 0.46 (0.36–0.60)), risk-based strategies (n = 11 studies, RR 0.65 (0.48–0.87)), or universal strategies (n = 16 studies, RR 0.37 (0.25–0.55)) was associated with a reduced risk of EOGBS infection compared to no strategy. In direct comparison, universal strategies were associated with a reduced risk of EOGBS compared to a risk-based strategy (n = 17 studies, RR 0.41 (0.30–0.55)), while the proportion of women receiving IAP did not differ between risk-based (16%) and universal (21%) strategies (n = 9 studies, RR 1.29 (0.95–1.75)). There was no antimicrobial resistance of EOGBS isolates to penicillin or ampicillin (n = 11 studies).

**Interpretation:**

Any IAP strategy could reduce the risk of EOGBS infection without evidence of increasing antimicrobial resistance. Universal strategies give the largest reduction in the EOGBS burden, while not exposing a significantly higher proportion of pregnancies to IAP compared to risk-based strategies.

**Funding:**

UNDP-UNFPA-UNICEF-WHO-World Bank Special Programme of Research, Development and Research Training in Human Reproduction, a cosponsored programme executed by the 10.13039/100004423World Health Organization.

**Research in context**

**Evidence before this study**

Early-onset Group B Streptococcal (EOGBS) infection affects all countries and leads to substantial morbidity and mortality. Intrapartum antibiotic prophylaxis (IAP) lowers risk of EOGBS infection, but strategies vary. Strategies include universal microbiology-based (universal) strategies, where IAP is administered to all women with presence of GBS colonisation, and risk-factor based (risk-based) strategies, where IAP is administered to women in the presence of risk factors (e.g. preterm labour). Previous systematic reviews and meta-analysis demonstrated that universal strategies may be more effective as compared to risk-factor based strategies, but there is no consensus on any optimal IAP strategy across multiple perinatal outcomes. In addition, several concerns related to IAP administration have been raised and include possible increases in non-GBS early-onset sepsis (EOS), antimicrobial resistance and the proportion of pregnancies exposed to IAP. Therefore, this review aimed to provide up-to-date evidence on the effectiveness and possible harm of various IAP strategies.

We searched MEDLINE, Embase and Web of Science for papers with no language or date restriction in May 2024, using the following search terms: “pregnancy” OR “newborn infant” AND “Group B streptococcus” AND “screening” OR “culture-based” OR “polymerase chain reaction” OR “risk-based” OR “prevention” OR “guidelines” OR “early onset”. The systematic search yielded 72 observation studies.

**Added value of this study**

This study extends previous systematic reviews in terms of size (including more than 10 million live births for meta-analysis) and the scope of included outcomes ((term) EOGBS, non-GBS early-onset sepsis, all early-onset sepsis, EOGBS-related mortality, IAP administration, antimicrobial resistance and maternal peripartum infection). Our findings show that implementation of any strategy reduces the risk of EOGBS, EOGBS-related mortality and non-GBS EOS compared to no strategy. Universal strategies lead to a larger reduction in EOGBS infection compared to risk-based strategies, with similar antibiotic exposure. Possible concerns related to increasing antimicrobial resistance of EOGBS isolates due to a general increase in antibiotic exposure after implementation of IAP strategies are not yet grounded for first-line agents (penicillin and ampicillin), but there is some evidence for increased resistance to alternative agents when penicillin allergy is present (erythromycin and clindamycin).

**Implications of all the available evidence**

This review serves as a basis for formulating evidence-based guidelines on IAP strategies. Findings demonstrate that all IAP strategies included in this review are likely effective with little explicit harm, but relevant contextual factors must be taken into account, including EOGBS burden, GBS colonisation rate, cost, feasibility, and providers' and women's views.

## Introduction

Early-onset Group B Streptococcus (EOGBS) infection, comprising sepsis, pneumonia and meningitis, are a leading cause of neonatal morbidity and mortality, with a worldwide incidence of 0.41 per 1000 live births and a mortality rate of 4–10% in high-income countries.^1^^,^^2^ EOGBS infections are defined as the presence of Group B Streptococcus (GBS) within 7 days of birth in normally sterile fluids, such as blood and cerebrospinal fluid.^3^^,^^4^ Infants may be infected antenatally by vertical transmission in colonised pregnant women.^5^ Globally, GBS colonisation of the recto-vaginal tract is present in 10–30% of all pregnant women during pregnancy.^6^ Therefore, prevention of the sequelae of GBS colonisation contributes importantly to perinatal health.

Currently, prevention of vertical transmission consists of administration of intrapartum antibiotic prophylaxis (IAP) at least 4 h before birth, which is associated with a reduced risk of EOGBS infection.^7^^,^^8^ IAP is administered according to various screening strategies applied in different settings. Risk-factor based screening (‘risk-based’) strategies are used in some settings, where IAP is administered according to the presence of any risk factor for EOGBS during pregnancy.^9^ In universal microbiology-based screening (‘universal’) strategies GBS colonisation is determined in all pregnant women antenatally and IAP is administered to women with GBS colonisation.^7^ In addition, IAP can be administered with any combination of elements from risk-based and universal strategies (‘other’).^10^^,^^11^

Previous systematic reviews and meta-analyses reported that universal strategies were associated with a lower incidence of EOGBS infection compared to risk-based strategies or having no strategy.^12^^,^^13^ However, IAP strategies still vary considerably around the world and are largely absent in developing countries. Reasons for the variation in IAP strategies include discussion concerning increased antibiotic use, antimicrobial resistance development and risk of a possible increase in non-GBS early-onset sepsis (EOS), caused by pathogens like *Escherichia coli*.^14^, ^15^, ^16^ Moreover, even when implementing universal strategies, there is no consensus of timing of GBS determination, though most guidelines recommend determination between 35 and 37 weeks’ gestation.

In order to contribute to consensus on GBS prevention strategies, this review elaborates on previous work, while incorporating up-to-date evidence on a variety of outcomes needed for evidence-based and nuanced policy-making. We evaluated the effectiveness of different prevention strategies, by comparing maternal and neonatal infectious morbidity and mortality, the frequency of IAP administration, and the presence of antimicrobial resistance. Furthermore, we evaluated timing of GBS determination in universal strategies to prevent EOGBS infection in newborns.

## Methods

### Protocol and registration

The Preferred Reporting Items for Systematic Reviews and Meta-Analyses (PRISMA) guidelines and Cochrane Handbook for Systematic Reviews were used to conduct and report this systematic review and meta-analysis.^17^^,^^18^ The protocol was registered in PROSPERO, with ID CRD42023411806.^19^

### Eligibility criteria

#### Study designs

We included any randomised and non-randomised study with human participants that reported on any of the outcomes of interest (specified below) when comparing/describing at least two different strategies for IAP administration to prevent EOGBS infections, including no strategy. Non-randomised studies included non-randomised interventional studies (e.g. quasi-randomised controlled trials) and uncontrolled observational studies (e.g. cohort studies). Studies were eligible regardless of sample size, year, country or language of publication and temporal data collection.

Studies were excluded when:

- only qualitative data was presented;
- strategies coincided;
- data was already published in other articles^h^;
- data was based on models, not actual cases;
- the full-text article could not be obtained, or;
- the article was categorised as case report, case series, letter to the editor, commentary, or conference abstract.

#### Participants

Participants were pregnant women and neonates.

#### Interventions

The intervention consisted of strategies for IAP administration (including no strategy). Risk-based strategies were defined as strategies where IAP was administered according to the presence of any risk factor for EOGBS during pregnancy, such as a history of an infant with EOGBS infection, presence of GBS bacteriuria during pregnancy, or presence of intrapartum fever, preterm labour or prolonged rupture of membranes >18 h.^9^ Universal strategies were defined as strategies where GBS colonisation was determined using microbiological testing and IAP was administered to all women positive for GBS colonisation.^7^ Other strategies were defined as strategies where IAP was administered based on any combination of elements from risk-based and universal strategies. ‘Other’ strategies usually consisted of strategies where both risk-based and universal strategies were implemented in parallel and selective strategies that only treat pregnant women positive for GBS colonisation with the presence of least one risk factor.^10^^,^^11^ Lastly, IAP was sometimes found to be administered without any official screening (‘no’) strategy on an individual basis and at the discretion of the attending physician.^12^^,^^20^

#### Outcome measures

Review outcomes were selected based on the critical and important outcomes used in the WHO recommendations on the prevention and treatment of maternal peripartum infections.^21^ Outcomes listed below were used as primary outcomes for the review. Protocol outcomes were specified into the following outcomes.

- Incidence of EOGBS infection per 1000 live births or pregnant women (as defined by study authors).
- Incidence of non-GBS early-onset sepsis (EOS) per 1000 live births or pregnant women.
- Incidence of all EOS per 1000 live births or pregnant women.
- EOGBS-related mortality rate per 1000 live births or pregnant women.
- EOGBS case-fatality rate (%).
- IAP administration rate (%).
- Antimicrobial resistance in EOS isolates (%).
- Maternal peripartum infection rate (%) (Supplementary File 2.7).

#### Information sources

Records were obtained through a systematic search of MEDLINE, Embase, and Web of Science databases. Additional publications were obtained manually by searching reference lists of relevant records and reviews.

#### Search strategy

The full search strategy used in MEDLINE is presented in Supplementary File 1. Search terms used included pregnancy, newborn, infant, Group B streptococcus, screening, culture-based, polymerase chain reaction, risk-based, prevention, guidelines, and early onset. In the final search, articles (regardless of language) were included until 2024. The last queries were run in May 2024.

#### Selection process

Potential studies were identified from the search strategy by double (independent) review (TJRP, GFH, TL, CA, YC, VB). Any disagreements between authors were resolved by an independent but unblinded third reviewer (TJRP, GFH, TL, CA, YC, VB) who was not involved in the disagreement. All titles and abstracts were placed in EndNote version X20 (Clarivate Analytics, Philadelphia, PA, USA) to automatically and manually deduplicate the list of studies. Afterwards, all articles were screened for eligibility via title and abstract in COVIDENCE (Veritas Health Innovation, Melbourne, Australia). Selected studies were subject to full-text screening. Full-text screening was done using aforementioned selection criteria.

#### Data collection process

Two authors (TJRP, GFH) independently extracted the following data: study design, study population and outcome measures using a preformed data extraction sheet.

#### Data items

Data items consisted of general information about the article, on the study population, IAP strategies, and outcome information. Outcome measures were manually calculated using raw data from the article. Missing data were reported as ‘No data’ in the tables.

#### Study risk of bias assessment

Risk of bias was assessed via the Risk of Bias in Non-randomised Studies of Interventions (ROBINS-I) tool.^22^ Studies were assessed by two independent reviewers (TJRP, GFH), and any discrepancies were handled through discussion until consensus. Seven domains of bias were assessed: confounding, selection of participants into the study, classification of interventions, deviations from intended intervention, missing data, measurement of outcomes and selection of the reported results. Each domain was scored as low, moderate, serious, critical, or unknown risk of bias. Confounding factors relevant to this review were specified before the studies were assessed for risk of bias.

#### Effect measures

EOGBS infection, non-GBS EOS and all EOS incidences were presented as cases per 1000 live births or pregnant women. EOGBS-related mortality rate was presented as deaths per 1000 live births or pregnant women. IAP administration incidence was presented as the percentage of pregnant women receiving IAP. Antimicrobial resistance incidence was presented as the percentage of resistance with respect to all EOS isolates examined. Incidence rates were calculated using the data provided in the studies.

Incidence of all early-onset infections and administration of IAP were compared between strategies with pooled risk ratios (RR) and 95%-Confidence intervals (CIs). EOGBS-related mortality rate (per 1000 live births or pregnant women), EOGBS case-fatality rate (%) and IAP administration rate (%) were also reported as a pooled incidence of the random-effects meta-analyses with a random intercept logistic regression model. Individual study 95%-CIs were reported with normal approximation confidence intervals. Meta-regression output was presented as β-coefficient and corresponding p-value.

#### Synthesis methods

Studies were only included in data synthesis if they reported on at least two different strategies (including no strategy) and if raw data was provided on the incidence of the outcomes of interest. The category ‘other strategies’ was not included in the meta-analysis due to heterogeneity in the strategies used, but these studies were included in the ‘any’ vs. ‘no’ strategy comparison. We used R version 4.2.1 (R: The R Project for Statistical Computing (r-project.org)) within Studio version 4.2.1 (R Studio, Boston, MA, USA, 2022) to combine studies, synthesize data (including forest plots), and create funnel plots. Heterogeneity was assessed by i) a Chi^2^-test for variation between studies and ii) the I^2^ statistic, which described the proportion of variation that is due to heterogeneity.

As we anticipated marked heterogeneity between studies that might influence treatment effect, in terms of study population, baseline infection incidence and timing of GBS determination, we performed subgroup analyses in addition to random-effects meta-regression. Subgroup analyses included comparisons in EOGBS infection incidence in studies that reported on term case incidence. Meta-regression analyses were performed post-hoc with an inverse variance method for all EOGBS comparisons that included at least 10 studies. We sought to delineate baseline EOGBS infection incidence (in cases per 1000 live births or pregnant women) and timing of GBS determination. Meta-regression analyses for timing of GBS determination were performed with two different methods in two different comparisons. In the comparison between universal and no strategy, the timing of GBS determination was compared between early determination before 33 weeks' gestation and late determination at 33–37 weeks' gestation. In the comparison between universal and risk-based strategies, timing of determination was compared between (antepartum) determination at 35–37 weeks’ gestation and (intrapartum) GBS colonisation determined within two days of birth.

We performed sensitivity analyses to assess the robustness of the synthesised results by excluding all studies with a serious or moderate-serious risk of bias in the comparisons for EOGBS infection incidence. Additionally, we performed leave-one out analyses for all EOGBS infection incidence comparisons. A p-value <0.05 was considered significant.

#### Reporting bias assessment

Publication bias analyses were carried out via visual inspection of the funnel plots and an Egger's test for funnel plot asymmetry for meta-analyses, including at least ten studies.^23^

### Certainty assessment

Certainty of the outcomes was assessed using the GRADE approach of the Cochrane Handbook for Systematic Reviews of Interventions.^24^ We critically evaluated study limitations, consistency of effect, imprecision, indirectness, and publication bias in the outcomes. An overall judgement of certainty assessment ranged from very low to high certainty evidence. Study limitations were evaluated with degree of risk of bias. Inconsistency was based on I^2^ and overlap of 95%-CI estimates. Indirectness was not relevant to this review. Imprecision was based on 95%-CI estimate of outcome. Publication bias was assessed as listed above.

### Ethics and informed consent

Ethical approval was not required for this evidence synthesis.

### Role of the funding source

This review was commissioned by the UNDP-UNFPA-UNICEF-WHO-World Bank Special Programme of HRP, SRH to inform WHO recommendations for Prevention and treatment of peripartum infections. Author TL is a staff of WHO, has access to the dataset and contributed to the decision to submit for publication.

## Results

### Study selection

**Fig. 1:**
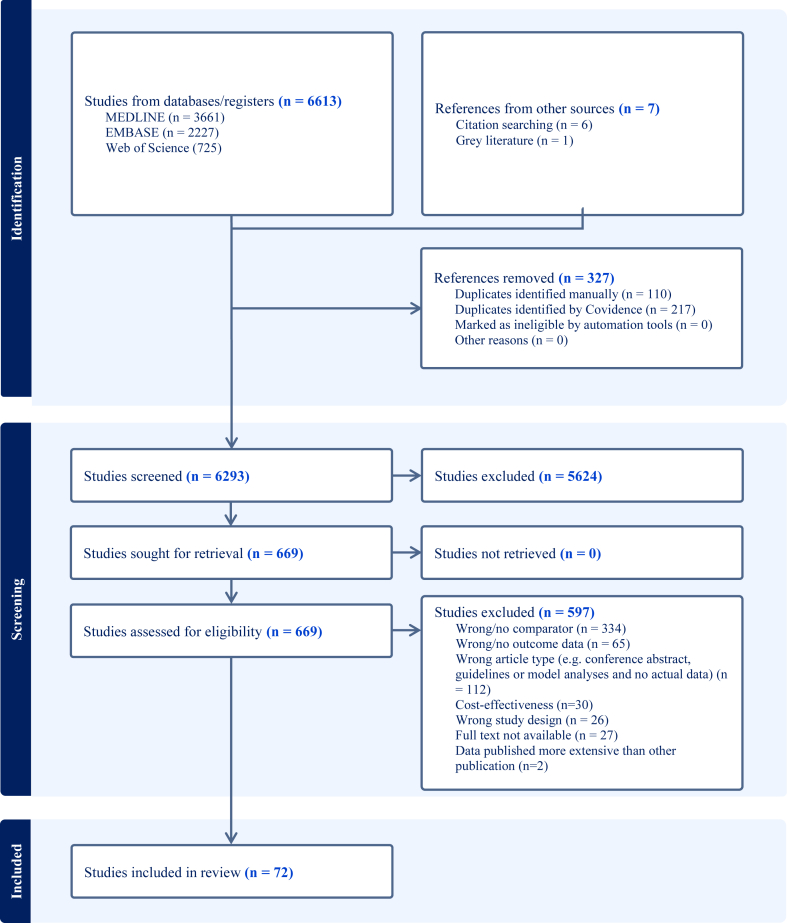
PRISMA study inclusion flowchart. PRISMA flowchart of retrieved and included studies.

The selection process is illustrated in a flow diagram in Fig. 1. The three databases overall provided 11,025 records, which were reduced to 6293 after removing duplicates. Title and abstract screening was done in all 6293 records: 5624 did not meet inclusion criteria, and the remaining 669 articles were reviewed in full-text. Ultimately, data on 82 articles^25^, ^26^, ^27^, ^28^, ^29^, ^30^, ^31^, ^32^, ^33^, ^34^, ^35^, ^36^, ^37^, ^38^, ^39^, ^40^, ^41^, ^42^, ^43^, ^44^, ^45^, ^46^, ^47^, ^48^, ^49^, ^50^^,^^51^, ^52^, ^53^, ^54^, ^55^, ^56^, ^57^, ^58^, ^59^, ^60^, ^61^, ^62^, ^63^, ^64^, ^65^, ^66^, ^67^, ^68^, ^69^, ^70^, ^71^, ^72^, ^73^, ^74^, ^75^, ^76^, ^77^, ^78^, ^79^, ^80^^,^^81^, ^82^, ^83^, ^84^, ^85^, ^86^, ^87^, ^88^, ^89^, ^90^, ^91^, ^92^, ^93^, ^94^, ^95^, ^96^, ^97^, ^98^, ^99^, ^100^, ^101^, ^102^, ^103^, ^104^, ^105^, ^106^ were presented and 72 articles^25^, ^26^, ^27^, ^28^, ^29^, ^30^, ^31^, ^32^, ^33^, ^34^, ^35^, ^36^, ^37^, ^38^, ^39^, ^40^, ^41^, ^42^, ^43^, ^44^, ^45^, ^46^, ^47^, ^48^, ^49^, ^50^^,^^51^, ^52^, ^53^, ^54^, ^55^, ^56^, ^57^, ^58^, ^59^, ^60^, ^61^, ^62^, ^63^, ^64^, ^65^, ^66^, ^67^, ^68^, ^69^, ^70^, ^71^, ^72^, ^73^, ^74^, ^75^, ^76^, ^77^, ^78^, ^79^, ^80^^,^^81^, ^82^, ^83^, ^84^, ^85^, ^86^, ^87^, ^88^, ^89^, ^90^, ^91^, ^92^, ^93^, ^94^, ^95^, ^96^ were included in the synthesis of the systematic review. Reasons for exclusions are listed in Fig. 1. No randomised controlled trials on this subject were found.

### Study characteristics

Overall, the 72 included studies included more than 10 million live births/deliveries from 20 different countries (Table 1). A total of 55 observational studies reported on EOGBS infection incidence, 18 on non-GBS EOS, 19 on all EOS, 2 on timing of GBS determination, 19 on EOGBS-related mortality, 16 on IAP administration and 16 on antimicrobial resistance. Two studies reported on timing of GBS determination. Study characteristics and results of individual studies are displayed in Table 1 and Supplementary File 2.

**Table 1:** Early-onset infection.

Author year	Study design	Time analysis	Country	Country income	Study setting/data source	IAP criteria: Universal strategies	IAP criteria: risk-based strategies	IAP criteria: other strategies	IAP agent	**Infection** definition	Incidence per 1000 live births or pregnant women (95%-CI)			
											No strategy	Risk strategy	Universal strategy	Other strategies
Abdelmaaboud & Mohammed 2011	Retrospective observational study	Historical	Qatar	High	Principal public hospital in State	IAP to: i) carriers of rectovaginal and urine GBS culture at 35–37 weeks' gestation, and ii) carriers of GBS culture at presentation for all high-risk cases (preterm labour (PTL), prolonged rupture of membranes (PROM), intrapartum fever (fever), clinical chorioamnionitis or GBS colonisation).	IAP to: PTL, PROM, fever, and a previous infant with GBS disease.		Ampicillin initially 2 g intravenously (IV) and 1 g every 6 h (h). Penicillin allergy, 900 mg clindamycin every 8 h.	GBS: <72 h blood or cerebrospinal fluid (CSF)	1.9 (no data)	0.57 (0.36–0.86)	0.48 (0.30–0.71)	
Al Luhidan et al., 2019	Retrospective cohort study	Historical	Saudi Arabia	High	Tertiary care hospital	CDC 2002: IAP to: i) carriers of rectovaginal GBS colonisation at 35–37 weeks' gestation, ii) previous baby with invasive GBS infection, iii) GBS bacteriuria, and iv) if unknown carrier state, presence of PTL, PROM and fever.	RCOG 2012: IAP to: PTL, PROM, fever, previous infant with invasive GBS infection, and GBS bacteriuria.		CDC 2002: Penicillin G 5 million units IV initially and 2.5–3 million units every 4 h. Ampicillin as alternative. Penicillin allergy not at high risk of anaphylaxis, cefazolin 2 g IV and 1 g every 8 h. Penicillin allergy high risk of anaphylaxis and susceptibility available, clindamycin 900 mg IV every 8 h, or erythromycin 500 IV every 6 h. If susceptibility not possible or unknown or isolates resistant to clindamycin or erythromycin, vancomycin 1 g IV every 12 h. RCOG 2012: Penicillin G 3 g IV initially and 1.5 g every 4 h. If allergy not risk of anaphylaxis, then cephalosporin (e.g. cefuroxime 1.5 g IV initially and 750 mg every 8 h). If allergy high risk of anaphylaxis, vancomycin 1 g every 12 h.	GBS: <7 days in sterile body site (blood, CSF, suprapubic catheter and lungs).		1.02 (0.59–1.66)	0.24 (0.15–0.36)	
Andreu et al., 2003	Retrospective cohort study	Historical	Spain	High	Ten hospitals, 5 public, 2 private and 3 with financing agreements with public sector	IAP to: carriers of GBS colonisation. Hospitals used different timing of determination, but became uniform near end of study and most determined GBS colonisation between 35 and 37 weeks' gestation.			NI.	GBS: <7 days in sterile product (blood, CSF, etc).	1.92 (1.34–2.67)		0.27 (0.098–0.58)	
Angstetra et al., 2007	Prospective cohort study	Historical	Australia	High	Tertiary obstetric unit		No information (NI).	Rectovaginal swab at 34–37 weeks gestation, IAP to i) carriers of rectovaginal GBS colonisation at 34–37 weeks' gestation, ii) PTL, fever previous infant with GBS infection, and GBS bacteriuria regardless of GBS status, and iii) if unknown carrier state, presence of PROM.	Penicillin G 1.2 g IV initially then 600 mg 4 h. Penicillin-sensitive individuals either cephalothin 2 g IV initially and 1 g every 6 h or clindamycin 600 mg IV. every 8 h.	GBS:^1^ Non-GBS:^2^ All:^3^ <7 days in blood or CSF, admission to neonatal unit for treatment with antibiotics and ventilation, and signs of sepsis.		0.84 (0.55–1.23)^1^ 0.94 (0.63–1.34)^2^ 1.78 (1.34–2.31)^3^		0 0.72 (0.32–1.61) 0.72 (0.32–1.61)
Bauserman et al., 2013	Retrospective cohort study	Historical	United States of America (USA)	High	Three hundred twenty-two NICUs managed by the Paediatric Medical Group	CDC 2002.			CDC 2002.	GBS: <3 days in blood, urine or CSF.	3.5 (no data)		2.6 (No data)	
Bekker et al., 2014	Retrospective nationwide surveillance study	Historical	The Netherlands	High	The Netherlands Reference Laboratory for Bacterial Meningitis			Dutch guidelines 1999: IAP to: i) previous infant with GBS infection, GBS bacteriuria or urinary tract infection and fever, and ii) GBS colonisation and PTL or PROM.	Dutch guidelines 1999: Penicillin G 5 million international units (IU) IV initially and 2.5 million IU every 4 h or 2 g of amoxicillin initially and 1 g every 4 h. Alternatively amoxicillin or ampicillin 2 g IV initially and 1 g every 4 h. Penicillin allergy, clindamycin 900 mg every 8 h. Alternatively, erythromycin 500 mg every 6 h.	GBS: <7 days in blood or CSF.	0.11 (0.10–0.13)			0.18 (0.16–0.20)
Björklund et al., 2017	Retrospective cohort study	Historical	Finland	High	Public financed tertiary delivery unity	IAP to: i) carriers of rectovaginal GBS polymerase chain reaction at admission, and ii) if carrier state unknown on presence of risk-factors.	IAP to: PROM, GBS bacteriuria, GBS colonisation, and previous infant with EOGBS.		Penicillin G 5 million IU IV initially and 2.5 million IU every 4 h. Penicillin allergy, cefuroxime 1.5 g IV initially and 750 mg every 8 h or clindamycin 900 mg IV every 8 h.	GBS: <3 days in clinical records databased with ICD code for GBS sepsis.		0.98 (0.20–2.87)	0	
Björnsdóttir et al., 2019	Retrospective and descriptive case study	Historical	Iceland	High	Iceland		IAP to: PTL, fever, PROM, and positive GBS cultures in late pregnancy or earlier deliveries.		NI.	GBS: <7 days.	0.7 (no data)	0.2 (no data)		
Brozanski et al., 2000	Retrospective observational study	Historical	USA	High	Tertiary referral hospital	CDC 1996 (similar to CDC 2002).		AAP 1992 and no strategy IAP to: i) carriers of rectovaginal GBS colonisation at 26–28 weeks' gestation or at admission with PTL, (premature) PROM, fever, multiple births, and ii) previous infant with invasive GBS infection.	Ampicillin, penicillin, clindamycin, or erythromycin.	GBS: <48 h in blood or CSF.			0.14 (0.038–0.36)	1.16 (0.81–1.60)
Chan et al., 2023	Retrospective cohort study	Historical	China	High-Middle	Eight public hospitals and 31 health centres	IAP to: i) carriers of rectovaginal GBS colonisation at 35–37 weeks' gestation, and ii) carrier state known before 35 weeks' gestation, GBS bacteriuria or history previous infant affected by GBS disease.	IAP to: previous infant with invasive GBS disease, PTL, GBS colonisation, and GBS bacteriuria.		Penicillin G 5 million IU IV initially and 2.5 million IU every 4 h. Penicillin allergy, erythromycin, clindamycin, or vancomycin according to sensitivity.	GBS^1^: Non-GBS^2^: All^3^: <8 days in blood or CSF.		1.03^1^ (0.87–1.22) 2.21^2^ (1.97–2.48) 3.25^3^ (2.95–3.57)	0.26^1^ (0.21–0.32) 2.00^2^ (1.85–2.16) 2.27^3^ (2.11–2.43)	
Cho et al., 2019	Retrospective cohort study	Historical	Taiwan	High	Tertiary obstetric unit	CDC 2010 (similar to CDC 2002).			Penicillin, ampicillin, or cefazolin.	GBS: <7 days in sterile site (blood, CSF or urine) with signs of clinical disease	2.8 (no data)		0 (no data)	
Clemens & Gable 2002	Retrospective cohort study	Historical	USA	High	Nonacademically affiliated community hospital	CDC 1996 (similar to CDC 2002).				EOGBS	1.93 (no data)		0.4 (no data)	
Darlow et al., 2016	Prospective and retrospective surveillance study	Historical	New Zealand	High	New Zealand Paediatric Surveillance Unit (all hospitals)		IAP to: PTL, PROM, fever, previous infant with GBS infection, and GBS bacteriuria.		Penicillin or amoxycillin.	GBS: <3 days in blood, CSF or pleura with clinical and laboratory evidence of spesis	0.50 (0.38–0.65)	0.23 (0.15–0.33)		
Eberly & Rajnik 2009	Retrospective cohort study	Historical	USA	High	Hospitals withing the Department of Defence	CDC 2002.		CDC 1996 IAP to: i) PTL, PROM and fever, ii) carriers of rectovaginal GBS screening at 35–37 weeks' gestation, iii) if unknown carrier state, presence of risk-factors, iv) previous infant with GBS infection, and v) GBS bacteriuria.	CDC 1996: Penicillin G 5 million IU IV initially and 2.5 million IU IV every 4 h. Alternatively, ampicillin 2 g IV initially and 1 g IV every 4 h. Penicillin allergy not at high risk of anaphylaxis, cefazolin 2 g IV initially and 1 g every 8 h. Penicillin allergy at high risk of anaphylaxis and susceptibility available, clindamycin 900 mg IV every 8 h, or erythromycin 500 IV every 6 h. If susceptibility not possible or unknown or isolates resistant to clindamycin or erythromycin, vancomycin 1 g IV every 12 h. CDC 2002.	GBS: <7 days with possession of diagnosis group codes and lack of other diagnosis group codes that refer to non-GBS bacteria.	1.95 (1.78–2.14)		0.47 (0.39–0.56)	0.72 (0.64–0.82)
Ecker et al., 2013	Retrospective cohort study	Historical	USA	High	Regional tertiary care centre	CDC 2002.	NI.		Penicillin or ampicillin.	GBS^1^: Non-GBS^2^: All^3^: ≤7 days in blood, urine or CSF with appropriate treatment.	2.06 (1.46–2.81)^1^ 3.48 (2.74–4.43)^2^ 5.54 (4.53–6.70)^3^	0.96 (0.51–1.64)^1^ 4.28 (3.32–5.53)^2^ 5.24 (4.09–6.60)^3^	1.11 (0.55–1.98)^1^ 3.33 (2.29–4.67)^2^ 4.44 (3.30–5.96)^3^	
Edwards et al., 2003	Retrospective cohort study	Historical	USA	High	General hospital	CDC 1996.			Ampicillin prior to march 1995 and penicillin thereafter.	GBS^1^: Non-GBS^2^: All^3^: <7 days in blood.		1.69 (0.92–2.83)^1^ 2.41 (1.47–3.72)^2^ 4.10 (2.84–5.73)^3^	1.01 (0.46–1.92)^1^ 3.60 (2.46–5.07)^2^ 4.62 (3.49–6.25)^3^	
Eisenberg 2005	Retrospective cohort study	Concurrent	USA	High	All acute care hospitals in four major counties of Tennessee	IAP to: carriers of GBS culture at least 2 days before delivery.	IAP to: no GBS culture <2 days and PTL, PROM, fever, GBS bacteriuria, or previous infant with invasive GBS disease.		NI.	GBS: <7 days in blood or CSF.		0.85 (0.56–1.25)	0.40 (0.19–0.74)	
Factor et al., 1998	Retrospective cohort study	Historical	USA	High	Non-profit, tertiary care hospital		ACOG 1992: IAP to: PTL, PROM, or fever.		Ampicillin, cefazolin, cefoxitin, vancomycin, cephalothin, erythromycin, and oxacillin	GBS: <7 days in blood or CSF.	1.70 (0.99–2.73)	0.15 (0.038–0.84)		
Freitas & Romero 2017	Retrospective cohort study	Historical	Brazil	High-Middle	Regional maternity hospital			Based on CDC 2010: IAP to i) carriers rectovaginal GBS colonisation at ≥24 weeks' pregnancy with PTL or ROM, ii) GBS bacteriuria and previous infant with GBS sepsis, and iii) if unknown carrier state, presence of PTL, PROM and fever (majority of patients).	Ampicillin 2 g IV initially and 1 g every 4 h. Penicillin allergy, clindamycin 900 mg IV every 8 h	GBS^1^: Non-GBS^2^: All^3^: <72 h in blood and antibiotic treatment ≥5 days or death <5 days while on antibiotic treatment.	0.51 (0.21–1.06)^1^ 1.40 (0.89–2.18)^2^ 1.91 (1.25–2.79)^3^			0^1^ 1.32 (0.71–2.45)^2^ 1.32 (0.71–2.45)^3^
Garland 1991	Retrospective observational study	Concurrent	Australia	High	Public teaching hospital	IAP to: carriers vaginal GBS culture at 32 weeks' gestation.			Penicillin 1 million IU IV every 6 h. Penicillin allergy, erythromycin 500 mg every 6 h.	EOGBS: in blood, CSF, surface swabs or urine	1.00 (0.66–1.46)		0.53 (0.30–0.86)	
Gibbs et al., 1994	Retrospective cohort study	Historical	USA	High	University hospital	IAP to: i) carriers of rectovaginal GBS culture at 26–28 weeks' gestation, ii) PTL, (premature) PROM, fever, chorioamnionitis with negative carrier status that turned positive, iii) previous infant with EOGBS infection, and iv) GBS bacteriuria.			Ampicillin 2 g IV initially and 1 g every 4 h. Penicillin allergy, erythromycin 500 mg every 6 h.	EOGBS sepsis:	1.50 (0.72–2.75)		1.03 (0.34–2.41)	
Gilson et al., 2000	Retrospective cohort study	Concurrent	USA	High	Academic medical centre	IAP to: i) carriers of GBS colonisation at 35–37 weeks' gestation, and ii) if unknown carrier state, presence of risk-factors.	IAP to: PTL, fever, PROM, and previous infant with GBS infection.		Before march 1995, ampicillin 2 g IV every 6 h. After march 1995, penicillin G 5 million IU initially and 2.5 million IU every 4 h. Penicillin allergy, clindamycin 900 mg every 8 h.	GBS: <7 days in blood, CSF, urine or suprapubic aspiration.		1.49 (0.41–3.81)	0	
Gopal Rao et al., 2017	Retrospective observational study	Historical	UK	High	General hospital	IAP to: carriers of rectovaginal GBS colonisation at 35–37 weeks' gestation	NICE 2012. IAP to: previous infant with GBS infection, GBS bacteriuria, GBS colonisation, and fever.		Penicillin G 3 g IV initially and 1.5 g every 4 h. Penicillin allergy, clindamycin.	GBS: <7 days in blood, CSF or other sterile fluids.		1.12 (0.78–1.57)	0.33 (0.068–0.96)	
Gosling et al., 2002	Questionnaire survey	Concurrent	New Zealand	High	Nineteen public hospitals			IAP to: i) carriers antenatal GBS culture, and ii) PTL, PROM, fever, previous infant with GBS infection, and GBS bacteriuria, and iii) a combination of strategy i) and ii).	Amoxicillin and penicillin. Penicillin allergy, erythromycin, clindamycin or cephalosporins.		1.44 (0.47–3.36)			0.46 (0.26–0.76)
Hafner et al., 1998	Retrospective and prospective cohort study	Historical	Austria	High	Sociomedical centre	IAP to: carriers of rectovaginal GBS culture at 33–35 weeks' gestation.	IAP to: (premature) PTL, PROM, fever, previous infant with GBS infection, and maternal diabetes mellitus.		Amoxicillin with clavulanic acid 2.2 g every 6 h. Penicillin allergy, 600 mg clindamycin.	GBS: in throat, umbilicus, ears or blood and signs of sepsis.		5.41 (3.31–8.34)	1.01 (0.28–2.59)	
Håkansson et al., 2017	Retrospective cohort study	Historical	Sweden	High	National health registers of all hospitals in Sweden		IAP to: PTL, PROM, fever, GBS bacteriuria, and previous infant with GBS infection.		NI.	GBS: <7 days in blood or CSF.	0.40 (0.33–0.48)	0.30 (0.24–0.36)		
Hong et al., 2019	Retrospective cohort study	Historical	South-Korea	High	Tertiary hospital	CDC 2010.	IAP to: PROM, fever, GBS bacteriuria, and previous infant with GBS sepsis.		Cefazolin 2 g IV initially and 1 g every 8 h. Cephalosporin allergy, ampicillin 2 g IV initially and 1 g every 4 h. Penicillin allergy, vancomycin and clindamycin.	GBS^1^: Non-GBS^2^: All^3^: <7 days in blood.		0.77 (0.019–4.28)^1^ 1.54 (0.19–5.54)^2^ 2.31 (0.48–6.72)^3^	0^1^ 0^2^ 0^3^	
Horváth et al., 2013	Prospective cohort study	Historical	Hungary	High	University hospital			IAP to i) carriers of rectovaginal GBS colonisation at 30–32 weeks' gestation, and ii) PTL, (premature) PROM, fever, multiple gestation, diabetes and polyhydramnios.	Ampicillin 2 g IV initially and 1 g ampicillin every 4 h. Penicillin allergy, erythromycin or clindamycin in equivalent dosage.	GBS: <7 days in blood or CSF with clinical signs of GBS.	1.57 (1.07–2.23)			0.31 (0.13–0.61)
Hung et al., 2018	Retrospective cohort study	Historical	Taiwan	High	National Health Insurance database (all hospitals)	IAP to: carriers of rectovaginal GBS colonisation at 35–37 weeks' gestation.				GBS: <7 days in hospital record with ICD-9 codes for GBS sepsis, meningitis and pneumonia.	1.0 (no data)		0.16 (0.10–0.23)	
Isaacs & Royle 1999	Prospective observational study	Historical	Australia	High	Australian neonatal units of the Australasian Study Group for Neonatal Infection			Different strategies in hospitals comprising no, risk-based, universal and other strategies.	Usually ampicillin or penicillin.	GBS^1^: Non-GBS^2^: All^3^: <48 h in blood, CSF or urine with clinical sepsis.	2.86 (2.22–3.60)^1^ 1.22 (0.86–1.75)^2^ 4.08 (3.32–4.96)^3^			0.54 (0.39–0.73)^1^ 0.50 (0.36–0.69)^2^ 1.04 (0.83–1.30)^3^
Jeffery & Moses Lahra 1998	Prospective cohort study	Historical	Australia	High	Tertiary referral hospital	IAP to: i) carriers of vaginal GBS colonisation at 28 weeks' gestation without risk-factors or 24 weeks if known risk-factor for preterm birth, ii) GBS bacteriuria, iii) previous infant with EOGBS infection, and iv) if unknown carrier state, presence of preterm labour.			Ampicillin 1 g every 6 h. Penicillin allergy, cephalosporin.	GBS: <48 h in blood and clinical signs of sepsis.	1.40 (0.60–2.75)		0.22 (0.095–0.43)	
Johansson Gudjónsdóttir et al., 2019	Retrospective cohort study	Historical	Sweden	High	University hospital, largest in Sweden		IAP to: PTL, PROM, fever, previous infant with EOGBS infection, and GBS bacteriuria.		NI.	GBS^1^: Non-GBS^2^: All^3^: <7 days in blood or CSF.	0.47 (0.34–0.64)^1^ 0.90 (0.72–1.12)^2^ 1.37 (1.13–1.64)^3^	0.44 (0.32–0.59)^1^ 0.48 (0.35–0.64)^2^ 0.92 (0.74–1.13)^3^		
Jourdan-da Silva et al., 2008	Retrospective observational study	Historical	France	High	Epibac; a network of hospital microbiology laboratories involved in monitoring invasive bacterial infections	IAP to: i) carriers of rectovaginal GBS colonisation at 35–37 weeks' gestation, and ii) if carrier state unknown, presence of PTL, (premature) PROM, previous infant with GBS infection and GBS bacteriuria.			NI.	GBS: <7 days.	0.69 (0.63–0.75) (no data)		0.23 (0.19–0.26) (no data)	
Katz et al., 1994	Retrospective cohort study	Historical	USA	High	Public academic tertiary care medical centre	IAP to: i) carriers of rectovaginal GBS culture at 24–28 weeks' gestation, ii) GBS bacteriuria, and iii) if carrier state unknown, presence of PROM and PTL.			Ampicillin 2 g IV every 6 h. Penicillin allergy, cefazolin 500 mg or clindamycin 600 mg every 6 h.	GBS: in blood	2.02 (0.55–5.17)		0	
Katz et al., 1999	Retrospective cohort study	Historical	USA	High	Urban tertiary centre			IAP to: i) carriers of rectovaginal GBS culture at 28 weeks' gestation and PTL, and ii) (premature) PROM, fever and previous infant with GBS infection.	Ampicillin 2 g IV every 6 h. Penicillin allergy, clindamycin 600 mg every 6 h.	GBS: <7 days in blood or CSF with clinical signs of sepsis.	2.15 (1.50–2.99)			2.19 (1.34–3.38)
Ko et al., 2021	Retrospective cohort study	Historical	Taiwan	High	Two referral hospitals with level 3 NICUs	IAP to: carriers of rectovaginal GBS colonisation at 35–37 weeks' gestation.	IAP to: PTL, PROM, fever, previous infant with invasive GBS infection and GBS bacteriuria.		Ampicillin, penicillin or cefazolin.	GBS^1^: Non-GBS^2^: All^3^: <3 days in blood or CSF.		0.62^1^ (no data) 1.44^2^ (no data) 2.06^3^ (no data)	0.38^1^ (no data) 1.01^2^ (no data) 1.39^3^ (no data)	
Lee et al., 2021	Retrospective cohort study	Historical	China	High-Middle	Tertiary university hospital	IAP to: i) carriers of rectovaginal GBS colonisation at 35–37 weeks' gestation.	IAP to: i) PTL, PROM and fever.		Ampicillin 2 g IV initially and 1 g every 4 h.	GBS: <7 days in blood.		0.27 (0.12–0.51)	0	
Levine et al., 1999	Retrospective observational study	Historical	USA	High	The Infection Control Surveillance Database			CDC 1996.	Ampicillin or cefoxitin.	GBS^1^: Non-GBS^2^: All^3^: <7 days in blood or CSF.	1.74 (1.17–2.48)^1^ 0.99 (0.61–1.59)^2^ 2.72 (2.00–3.62)^3^			0^1^ 2.14 (1.07–4.28)^2^ 2.14 (1.07–4.28)^3^
Lin et al., 2011	Retrospective cohort study	Historical	Taiwan	High	Private hospital	IAP to: i) carriers of rectovaginal GBS culture at 35–37 weeks' gestation, ii) GBS bacteriuria, iii) previous infant with GBS infection, and iv) if unknown carrier state, presence of PTL, PROM and fever.			Ampicillin 2 g IV initially and 1 g every 4 h. Alternatively, penicillin G. Ampicillin allergy, cefazolin. Penicillin allergy, clindamycin or erythromycin.	GBS^1^: Non-GBS^2^: All^3^: <72 h in blood.	0.66 (0.32–1.21)^1^ 0.79 (0.45–1.39)^2^ 1.45 (0.91–2.19)^3^		0.23 (0.063–0.59)^1^ 0.92 (0.53–1.49)^2^ 1.15 (0.74–1.78)^3^	
Locksmith et al., 1999	Retrospective cohort study	Historical	USA	High	University tertiary care centre	CDC 1996.		Until 1993, IAP to: carriers of GBS colonisation at hospital with PTL, premature PROM and another risk-factor (unspecified).	Until 1995, ampicillin 2 g IV initially and 1 g every 6 h. After 1995, penicillin 5 million IU and 2.5 million IU every 4 h. Amoxicillin for premature PROM and GBS colonisation.	GBS: <7 days in blood or CSF.			1.35 (0.49–2.93)	2.95 (1.87–4.42)
Locksmith et al., 1999	Retrospective cohort study	Historical	USA	High	University tertiary care centre	CDC 1996.		After 1993, ACOG guidelines but unknown which ones. IAP to: i) if unknown carrier state, presence of PTL, PROM, fever, and previous infant with GBS sepsis, and ii) strategy above.	Until 1995, ampicillin 2 g IV initially and 1 g every 6 h. After 1995, penicillin 5 million IU and 2.5 million IU every 4 h. Amoxicillin for premature PROM and GBS colonisation.	GBS: <7 days in blood or CSF.			1.35 (0.49–2.93)	1.90 (1.06–3.12)
López Sastre et al., 2005	Prospective surveillance study	Historical	Spain	High	Twenty eight acute care teaching hospitals	IAP to: i) carriers of rectovaginal GBS culture at 35–37 weeks' gestation, ii) GBS bacteriuria, and iii) previous infant with GBS infection.			Societies of Clinical Microbiology and Infectious Disease and Chemotherapy 1998: Ampicillin 2 g IV initially and 1 g every 4 h or penicillin G 5 million IU IV initially and 2.5 million IU every 4 h. Beta-lactam allergy, clindamycin 900 mg every 8 h, or erythromycin 500 mg every 6 h.	GBS^1^: Non-GBS^2^: All^3^: <3 days in blood with one clinical sign and at least one laboratory abnormality consistent with infection.	1.11 (0.89–1.37)^1^ 1.11 (0.89–1.37)^2^ 2.22 (1.90–2.57)^3^		0.70 (0.54–0.90)^1^ 1.03 (0.83–1.26)^2^ 1.73 (1.48–2.02)^3^	
Lu et al., 2022	Retrospective cohort study	Historical	Taiwan	High	University hospital with a level 3 NICU	IAP to: carriers of rectovaginal GBS culture at 35–37 weeks' gestation.			NI.	GBS: <72 h in blood or CSF.	1.1 (no data)		0.32 (no data)	
Lukacs & Schrag 2012	Cross sectional study	Historical	USA	High	National Centre for Health Statistics' National Hospital Discharge Survey data	CDC 2002 IAP to: i) carriers of rectovaginal GBS culture at 35–37 weeks' gestation, ii) previous baby with invasive GBS infection, iii) GBS bacteriuria, and iv) if GBS carrier rate unknown, PTL, PROM and fever. ACOG 2002 endorses CDC 2002 guidelines		CDC 1996. AAP 1997 IAP to: i) GBS bacteriuria, ii) previous infant with GBS infection, iii) carriers of rectovaginal GBS culture at 35–37 weeks' gestation, iv) if unknown carrier state, presence of risk-factors, and iv) PTL, PROM and fever. ACOG 1996 guidelines endorse CDC 1996.	CDC 1996, ACOG 1996 (penicillin), ACOG 2002, CDC 2002, and AAP 1997: Penicillin G 5 million IU IV initially and 2.5 million IU every 4 h. Alternatively, ampicillin 2 g IV initially and 1 g every 4 h. Penicillin allergy, clindamycin or erythromycin IV.	All: <7 days with ICD-9 codes for infection in perinatal period or septicaemia.	14.51 (14.28–14.74)		10.30 (10.05–10.56)	11.40 (11.17–11.64)
Main & Slagle 2000	Prospective observational study	Historical	USA	High	Tertiary perinatal referral centre	CDC 1996 = ACOG 1996. IAP to: i) carriers of rectovaginal GBS colonisation at 35–37 weeks' gestation, ii) term patients with fever >38 °C, iii) previous infant with GBS infection, and iv) if unknown carrier state, presence of PTL.	ACOG 1992 and CDC 1996. IAP to: PTL, (premature) PROM, fever, previous infant with EOGBS infection, and GBS bacteriuria.		ACOG 1992 and CDC 1996, but in reality: Ampicillin 2 g IV initially and 1 g every 4 h. Penicillin allergy, 900 mg clindamycin every 8 h.	GBS^1^: Non-GBS^2^: All^3^: <7 days in blood or CSF.	1.17 (0.51–2.31)^1^ 1.17 (0.51–2.31)^2^ 2.35 (1.34–3.80)^3^	1.13 (0.63–1.86)^1^ 0.30 (0.082–0.77)^2^ 1.43 (0.86–2.24)^3^	0.071 (0.0018–0.40)^1^ 0.35 (0.12–0.83)^2^ 0.43 (0.19–0.95)^3^	
Matsubara et al., 2013	Retrospective nationwide questionnaire surveillance	Historical	Japan	High	One hundred fifty-four hospital, 14 managed only outborn infants in absence of obstetric department, 62 NICUs and 76 regional centres	IAP to: i) carriers of GBS colonisation at 33–37 weeks' gestation except for elective caesarean deliveries, ii) previous infant GBS disease, and iii) if unknown carrier state, at delivery irrespective of gestational age.			NI.	GBS: <7 days in blood, CSF or joint aspirate.	0.075 (0.048–0.11)		0.098 (0.052–0.17)	
Matsubara et al., 2007	Multicentre questionnaire survey		Japan	High	Twenty-eight regional hospitals with 9 NICUs	23 hospitals routine antenatal determination (likely 33–37 weeks' gestation in Japan according to JSOG guidelines).			Ampicillin, piperacillin, or cefotiam. Loading doses of antibiotics varied from 0.5 to 2 g and interval differed from every 4 h until every 12 h.	EOGBS	0.11 (0.013–0.40)		0.10 (0.033–0.24)	
Ma et al., 2018	Retrospective cohort study	Historical	China	High-Middle	Eight public hospitals and 27 maternal and child health centres	IAP to carriers of rectovaginal GBS colonisation at 35–37 weeks' gestation.	IAP to: PTL, PROM, fever, previous infant GBS disease, and GBS bacteriuria.		NI.	GBS: <7 days in blood or CSF.		1 (0.7–1.1) (no data)	0.24 (0.16–0.34)	
O'Sullivan et al., 2019	Prospective active national surveillance study	Historical	UK and Ireland	High	Active surveillance (all paediatricians) and laboratory databases (all)		RCOG 2003: IAP to: PTL, fever, previous infant with GBS infection, GBS bacteriuria, GBS colonisation, and suspected chorioamnionitis. NICE 2012		Penicillin G, cephalosporin or vancomycin.	GBS: <7 days in blood, CSF or joint fluid.	0.48 (0.43–0.53)	0.57 (0.52–0.62)		
Petersen et al., 2014	Retrospective cohort study	Historical	Denmark	High	Public referral centre		Denmark recommendation 2004: IAP to: PTL, PROM, fever, GBS bacteriuria, and previous infant with invasive GBS infection.		NI.	GBS: <7 days in blood, CSF or tracheal swab.	2.04 (1.09–3.48)	1.50 (0.99–2.18)		
Phares et al., 2008	Retrospective cohort study	Historical	USA	High	Database Active Bacterial Core surveillance/Emerging Infections Program Network	CDC 2002.		CDC 1996, ACOG 1996, AAP 1997.	CDC 1996, ACOG 1996, AAP 1997, CDC 2002.	GBS: <7 days in blood or CSF.			0.34 (0.31–0.37)	0.47 (0.44–0.51)
Poulain et al., 1997	Prospective cohort study	Historical	France	High	University hospital regional reference century			IAP to: i) carriers of rectovaginal GBS colonisation at 28 weeks' gestation, GBS bacteriuria and one of the following risk-factors: PTL, PROM, fever, twin pregnancies, maternal diabetes, and ii) previous infant with GBS.	Amoxicillin 2 g IV initially and 1 g every 4 h.	GBS: Immediate in blood or CSF.	4.48 (1.65–9.73)			1.63 (0.44–4.17)
Puopolo & Eichenwald 2010	Retrospective cohort study	Historical	USA	High	University hospital	IAP to: carriers of rectovaginal GBS colonisation at 35–37 weeks' gestation.	IAP to: PTL, PROM, fever.		Ampicillin or clindamycin for risk-factor strategy. Penicillin G for universal strategy. Penicillin allergy, clindamycin, cefazolin, erythromycin and vancomycin.	GBS^1^: Non-GBS^2^: All^3^: <72 h in blood and neonatologist considered infant infected. Surviving infants treated with appropriate course of antibiotics ≥7 days.	2.16 (1.66–2.80)^1^ 1.54 (1.13–2.10)^2^ 3.72 (3.00–4.52)^3^	1.12 (0.79–1.53)^1^ 1.12 (0.79–1.53)^2^ 2.24 (1.77–2.79)^3^	0.43 (0.32–0.58)^1^ 1.16 (0.96–1.38)^2^ 1.59 (1.36–1.86)^3^	
Renner et al., 2006	Retrospective observational study	Historical	Switzerland	High	University hospital			IAP to: i) carriers of GBS screening at 35–37 weeks + PROM, fever or PTL, ii) unknown carriers state when risk-factor present, and iii) previous infant with GBS sepsis.	NI.	GBS: <7 days in blood.	0.99 (0.57–1.61)			0.53 (0.17–1.24)
Rottenstreich et al., 2019	Retrospective cohort study	Historical	Israel	High	University affiliated medical centre	IAP to: i) carriers of rectovaginal GBS colonisation at 35–37 weeks' gestation, and ii) if unknown carrier state, presence of risk-factors.	IAP to: PTL, PROM, fever, previous infant EOGBS, and GBS bacteriuria.		NI.	GBS: <7 days in normally sterile site.		0.36 (0.21–0.57)	0.19 (0.12–0.30)	
Sagrera et al., 2001	Retrospective cohort study	Historical	Spain	High	Private hospital	IAP to: i) carriers of rectovaginal GBS colonisation at 35–37 weeks' gestation, ii) unknown carrier state, and iii) previous child GBS and GBS bacteriuria during pregnancy		IAP to: i) carriers of intrapartum GBS colonisation with PTL, PROM, fever, GBS bacteriuria and previous infant with GBS infection.	Ampicillin 2 g IV initially and 1 g every 4 h. Penicillin allergy, erythromycin or clindamycin.	GBS: <7 days in blood, CSF or in urine with clinical symptoms.			1.12 (0.54–2.06)	2.44 (1.53–3.69)
Sakata et al., 2012	Retrospective cohort study	Historical	Japan	High	Public hospital	JSOG and JAOG 2008 IAP to: i) carriers of rectovaginal GBS colonisation at 33–37 weeks' gestation, ii) earlier infant GBS infection, and iii) unknown carrier state.			Sulbactam or ampicillin 1.5 g.	GBS: <3 days in blood or CSF with signs of infection.	0.51 (0.10–1.48)		0.40 (0.010–2.23)	
Schrag et al., 2002	Retrospective cohort study	Concurrent	USA	High	Multiple hospitals of the Emerging Infections Program Network	IAP to: carriers of GBS culture at least 2 days before delivery.	IAP to: all pregnant women without documentation of test for GBS with PTL, PROM, fever, previous infant with GBS infection, and GBS bacteriuria.		NI.	GBS: in normally sterile site.		0.68 (0.59–0.78)	0.33 (0.27–0.39)	
Share et al., 2001	Retrospective cohort study	Historical	USA	High	University hospital			AAP 1997, ACOG 1996.	AAP 1997, ACOG 1996.	GBS: <72 h in blood or CSF.	2.66 (1.62–4.10)			0.70 (0.33–1.28)
Simetka et al., 2010	Retrospective cohort study	Historical	Czech Republic	High	University hospital	IAP to: i) carriers of rectovaginal GBS colonisation at 35–37 weeks, ii) GBS bacteriuria, iii) previous infant with invasive GBS, iii) if unknown carrier state, presence of PTL, PROM, fever or positive rapid test for GBS colonisation.			Penicillin G 5 million IU IV and 2,5-3 million IU every 4 h. Alternatively, ampicillin 2 g IV initially and 1 g every 6 h. Penicillin allergy not at high risk of anaphylaxis, cephalosporins (cefazolin and cephalothin) 2 g IV initially and 1 g every 8 h. Penicillin allergy at high risk of anaphylaxis, clindamycin 900 mg IV every 8 h or vancomycin 1 g IV every 12 h.	GBS: <7 d in blood or CSF with clinical symptoms.	0.84 (0.17–2.45)		0.61 (0.13–1.79)	
Sorg et al., 2021	Retrospective cohort	Historical	Germany	High	The scientific network of the Barmer Ersatzkasse (insurance providers) 10% of population of Germany	IAP to: i) carriers of rectovaginal GBS culture at 35–37 weeks' gestation. or ii) if carrier state unknown, presence of risk-factors.		IAP to 2 strategies: i) carriers of rectovaginal GBS culture at 35–37 weeks' gestation, and ii) PROM, PTL, fever, previous infant with GBS infection, and GBS bacteriuria.	NI.	GBS: <72 h in blood, maternal cervical swab or other swabs.			0.5 (no data)	1.5 (no data)
Sridhar et al., 2014	Retrospective cohort study	Historical	India	Low-Middle	Tertiary level neonatal unit of teaching hospital		IAP to: PTL, (premature) PROM, fever, 3 or more gloved per vaginal examinations, chorioamnionitis, and GBS bacteriuria.		Ampicillin.	EOGBS	0.86 (0.59–1.13) (no data)	0.55 (0.36–0.72) (no data)		
Sutkin et al., 2005	Retrospective cohort study	Historical	USA	High	Tertiary referral hospital	CDC 1996.		AAP 1992 IAP to: carriers of rectovaginal GBS colonisation at 26–28 weeks' gestation with one of the following risk-factors: PTL (premature) PROM, fever, previous infant with EOGBS disease, and GBS bacteriuria. Before 1993, there was no implemented strategy.	Penicillin and clindamycin.	Non-GBS^2^: All^3^: <48 h in blood, CSF or other tissue.			1.11 (0.76–1.57)^2^ 1.25 (0.90–1.74)^3^	1.16 (0.83–1.60)^2^ 1.77 (1.36–2.30)^3^
Tapia et al., 2007	Retrospective cohort study	Historical	Chile	High	University hospital	IAP to: i) carriers of GBS colonisation at 35–37 weeks' gestation, ii) previous infant with invasive GBS disease, iii) GBS bacteriuria, and iii) if carrier state unknown, presence of PTL, PROM and fever.			NI.	GBS^1^: Non-GBS^2^: All^3^: <72 h in blood.	1.36 (0.54–2.79)^1^ 1.16 (0.52–2.58)^2^ 2.51 (1.34–4.30)^3^		0.11 (0.0028–1.79)^1^ 0.89 (0.38–1.75)^2^ 1.00 (0.52–1.92)^3^	
Towers & Briggs 2002	Prospective cohort study	Historical	USA	High	Non-profit hospital		IAP to: i) PTL, PROM and fever, and ii) carriers of inconsistent screening at 28 weeks' gestation or first prenatal visit.	IAP to: carriers of GBS colonisation at 35–37-weeks’ gestation, or IAP to: PTL, PROM, fever and; ii) carriers of inconsistent GBS screening at 28 weeks' gestation or first prenatal visit.	Until 1998 ampicillin. After 1998, penicillin G. Also use of cefazolin and cephalexin in preterm population. Penicillin allergy, erythromycin and/or clindamycin.	GBS^1^: Non-GBS^2^: All^3^: <7 days in blood with clinically symptomatic infant.	1.53 (1.10–2.07)^1^ 0.67 (0.32–1.41)^2^ 1.44 (0.81–2.38)^3^	0.36 (0.14–0.74)^1^ 1.02 (0.63–1.58)^2^ 1.38 (0.91–2.01)^3^		0.50 (0.24–0.92)^1^ 0.80 (0.49–1.31)^2^ 1.31 (0.89–1.92)^3^
Trollfors et al., 2022	Retrospective observational study	Historical	Sweden	High	All live births in southwest Sweden		IAP: fever, PTL, PROM, previous infant with invasive GBS infection, and GBS bacteriuria.		Penicillin.	GBS: <7 days.	0.73 (no data)	0.34 (no data)		
Uy et al., 2002	Retrospective population survey	Historical	USA	High	Tertiary care centre, division of university hospital.		CDC 1996. Adhere to risk-based guidelines, but prescription left to individual practitioner.	AAP 1992, ACOG 1992.	AAP 1992: Ampicillin 2 g IV initially, 1–2 g every 4–6 h or penicillin G 5 million IU every 6 h. Penicillin allergy, clindamycin or erythromycin. Also ACOG 1992 and CDC 1996.	GBS: <7 days in blood or CSF.	1.5 (no data)	0.29 (no data)		0.67 (no data)
van den Hoogen et al., 2010	Retrospective cohort study	Historical	Netherlands	High	University hospital level 3 NICU unit			Dutch guidelines 1999.	Dutch guidelines 1999.	Non-GBS^2^: All^3^: <48 h in blood and clinical signs of infection.	0.28 (0.23–0.34)^2^ 0.54 (0.46–0.62)^3^			0.10 (0.061–0.18)^2^ 0.23 (0.16–0.33)^3^
van Dyke et al., 2009	Retrospective cohort study	Historical	USA	High	Active Bacterial Core surveillance system 10 US states	Any documented colonisation prenatally or at admission performed 2 days or more before delivery, IAP to carriers of GBS colonisation.		Schrag et al., 2002: IAP to: i) carriers of GBS culture at least 2 days before delivery, and ii) all pregnant women without documentation of test for GBS with following risk-factors: PTL, PROM, fever, previous infant with GBS infection, and GBS bacteriuria.	Penicillin, ampicillin, cefazolin, clindamycin or vancomycin.	GBS: <7 days in normally sterile site.			0.31 (0.27–0.35)	0.50 (0.44–0.55)
Vergani et al., 2002	Retrospective cohort study	Historical	Italy	High	Tertiary referral centre		IAP to: PTL, PROM, fever, previous infant with GBS infection, and GBS bacteriuria.	IAP to: i) carriers of rectovaginal GBS culture at 26–28 and 35–37 weeks' gestation, and ii) PTL, PROM, fever, GBS bacteriuria, previous infant with GBS infection.	Ampicillin 2 g IV initially and 1 g every 4 h. Penicillin allergy, erythromycin 500 mg every 6 h.	GBS^1^: Non-GBS^2^: All^3^: <7 days in blood, CSF, auricular or pharyngeal culture with at least two inflammatory indices.	0.93 (0.40–1.84)^1^ 1.28 (0.71–2.32)^2^ 2.22 (1.33–3.46)^3^	0.78 (0.34–1.53)^1^ 0.87 (0.40–1.66)^2^ 1.65 (0.96–2.64)^3^		0.44 (0.16–0.95)^1^ 0.65 (0.34–1.26)^2^ 1.09 (0.66–1.81)^3^
Wicker et al., 2019	Prospective surveillance study	Historical	Germany	High	German Paediatric Surveillance Unit/Survey of Rare Diseases and Robert Koch Institute	IAP to: i) carriers of rectovaginal GBS culture at 35–37 weeks' gestation, and ii) if carrier state unknown, presence of risk-factors.		IAP to 2 strategies: i) carriers of rectovaginal culture at 35–37 weeks' gestation, or ii) PTL, PROM, fever, previous infant with GBS infection, and GBS bacteriuria.	NI.	GBS: <7 days in blood or CSF.			0.069 (0.055–0.084)	0.14 (0.12–0.16)
Yücesoy et al., 2004	Prospective, quasi-experimental study	Concurrent	Turkey	High-vMiddle	Antenatal clinic (in- and out-patient)	IAP to: i) carriers of rectovaginal GBS culture at 35–37 weeks' gestation, and ii) if unknown carrier state, presence of risk-factors.	CDC 1996.		Ampicillin 2 g IV initially and 1 g every 4 h.	GBS: <72 h in blood.		3.33 (0.69–9.71)	5.00 (0.13–27.54)	

When there was data available on more groups of infection: 1refers to the incidence of early-onset GBS infection; 2refers to the incidence of early-onset non-GBS infection, and; 3refers to the incidence of all early-onset infections.

### Risk of bias within studies

Overall risk of bias was critical in 3 studies, serious in 43 studies, moderate-serious in 3 studies, moderate in 22 studies and low in 1 study (Supplementary File 3). Many studies had problems in the domain of confounding due to a lack of data on the demographics for the total population or issues with retrospective outcome measurements, which were not standardised.

### Synthesis of results

#### EOGBS infection

**Fig. 2:**
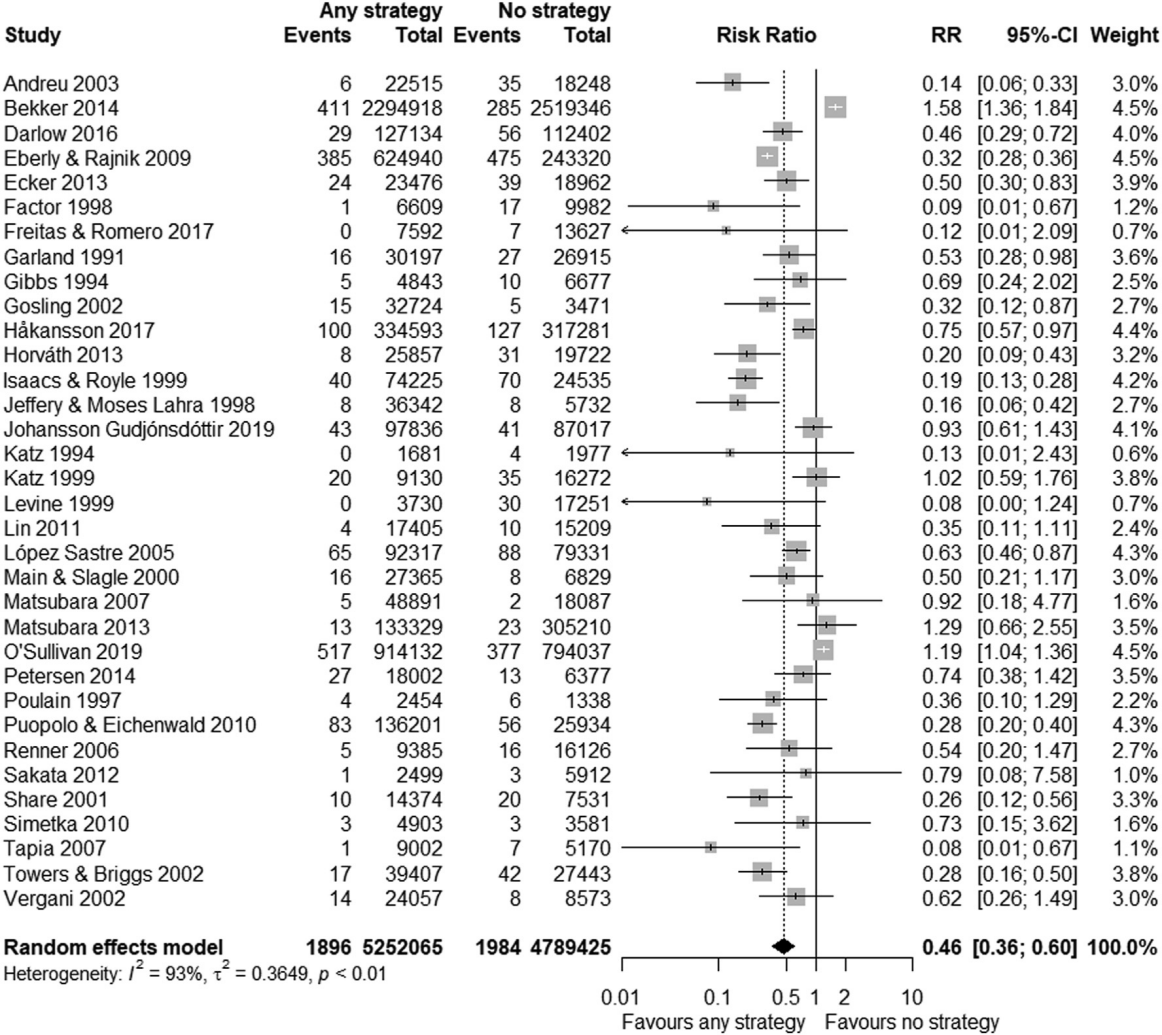
Forest plot of EOGBS infection: any vs no strategy. Forest plot of risk ratio (with 95%-CI) of EOGBS infection in any strategy versus no strategy.

**Fig. 3:**
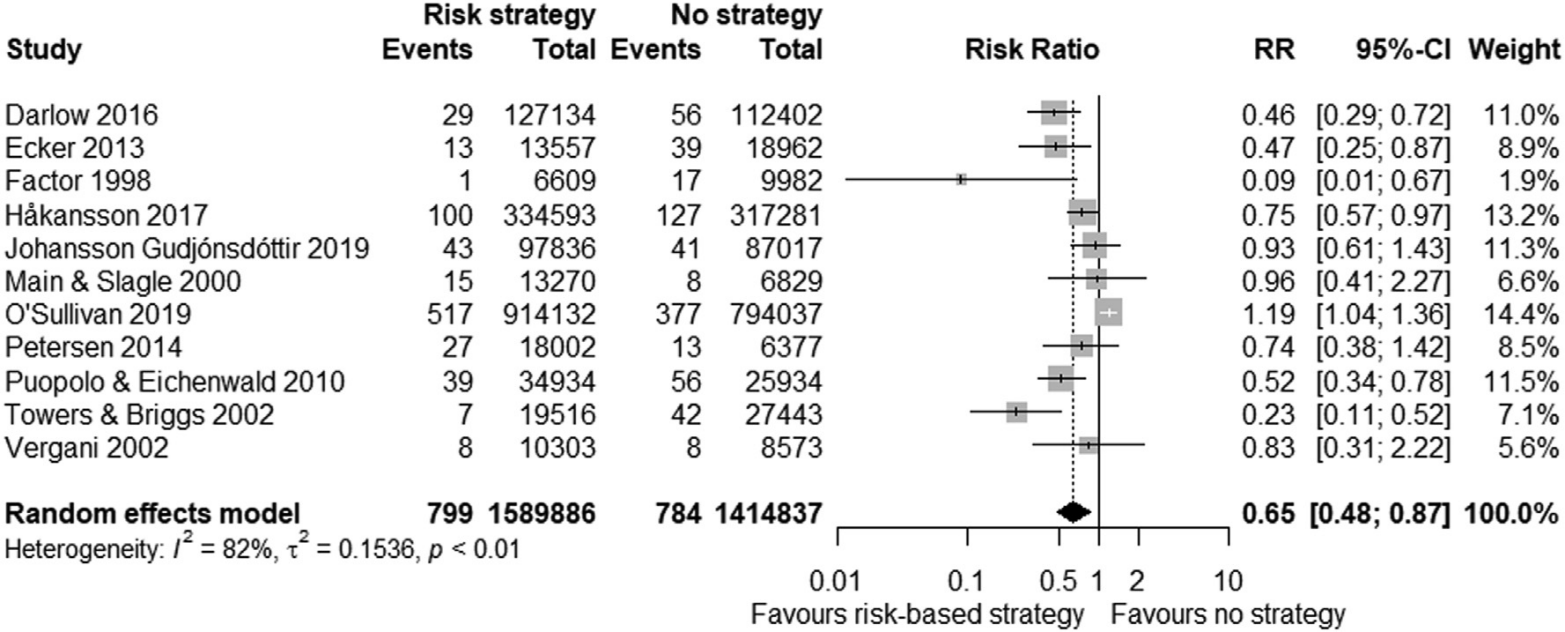
Forest plot of EOGBS infection: risk vs. no strategy. Forest plot of risk ratio (with 95%-CI) of EOGBS infection in risk-based strategies versus no strategy.

**Fig. 4:**
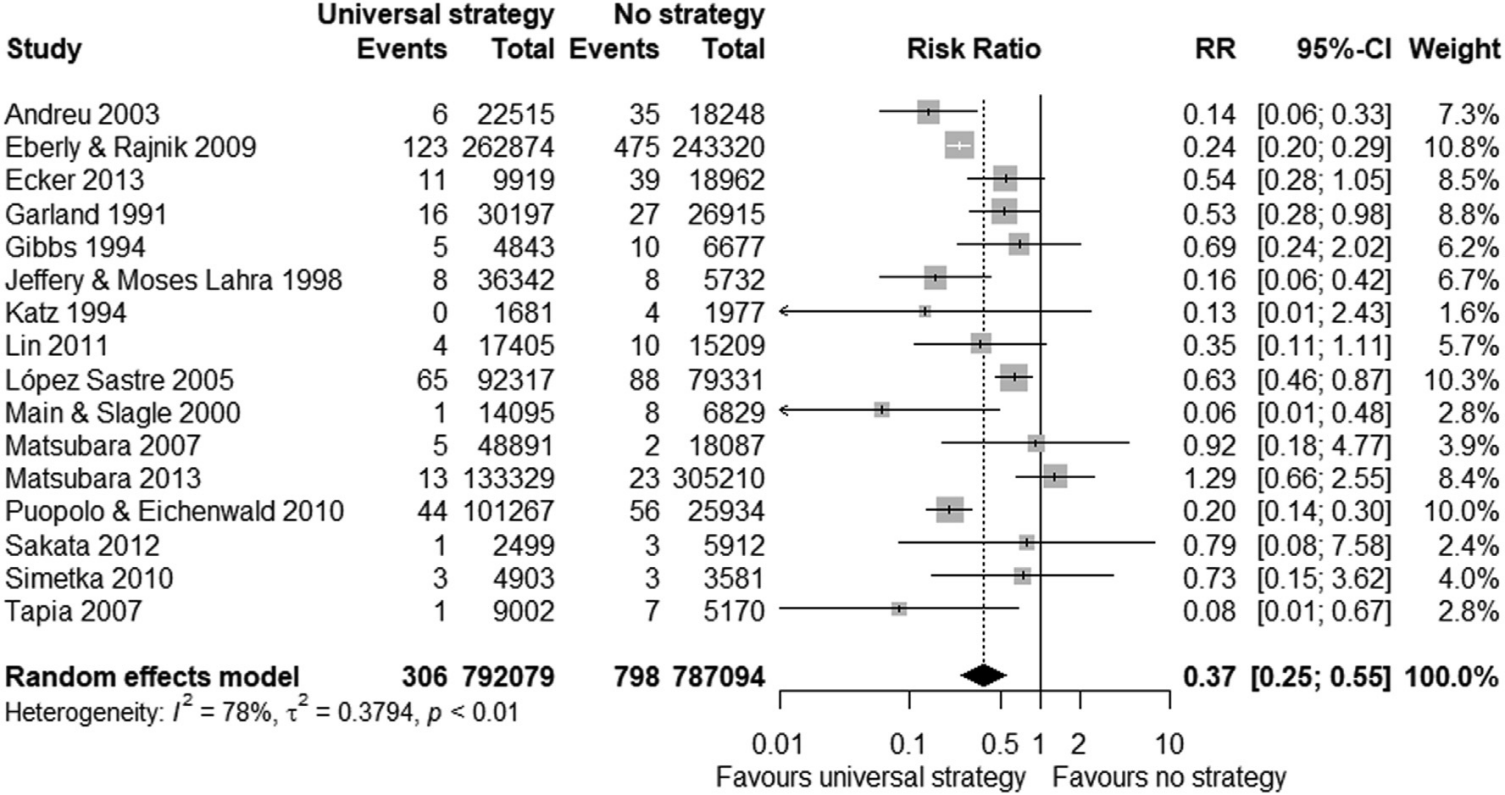
Forest plot of EOGBS infection: universal vs. no strategy. Forest plot of risk ratio (with 95%-CI) of EOGBS infection in universal strategies versus no strategy.

**Fig. 5:**
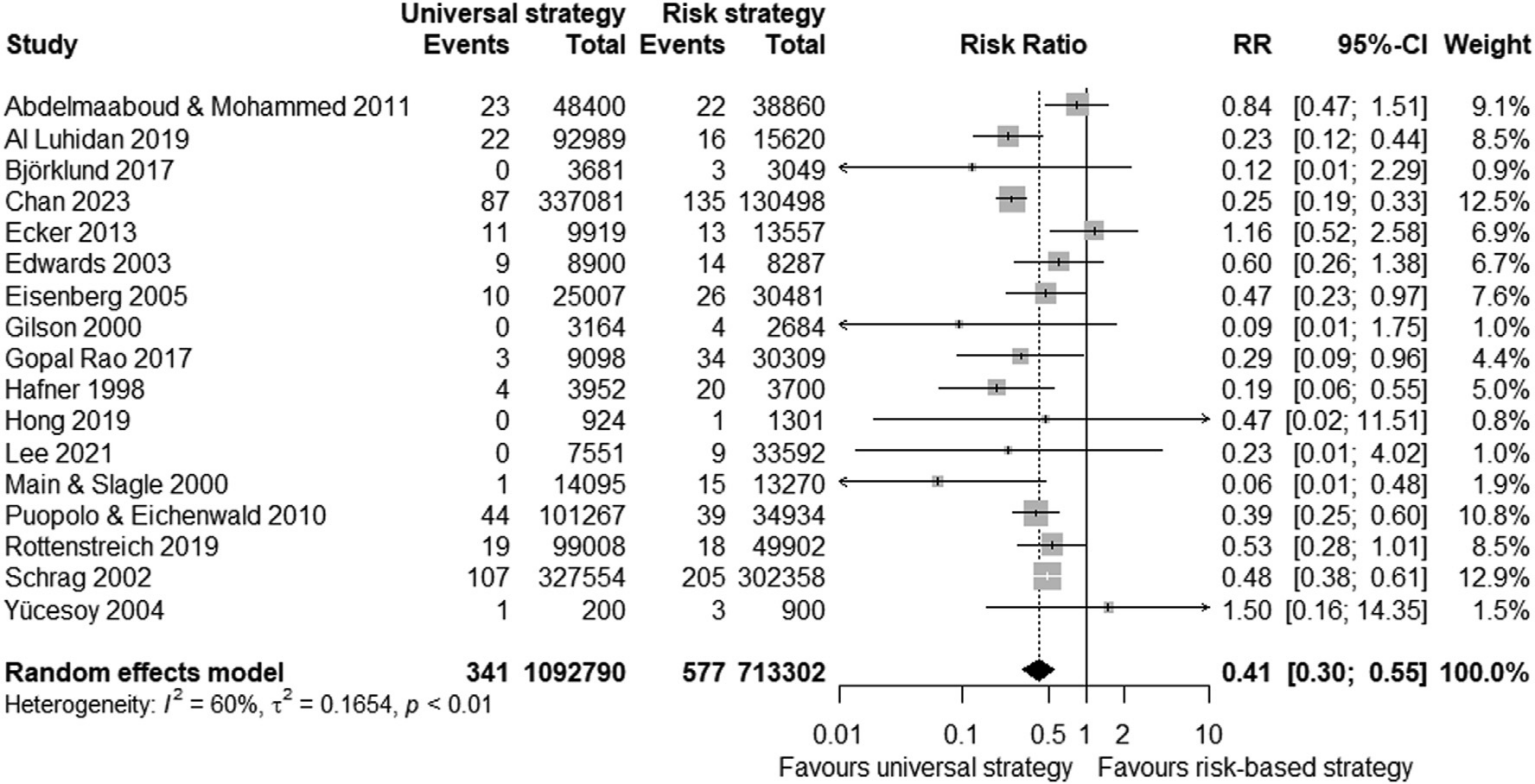
Forest plot of EOGBS infection: universal vs. risk strategies. Forest plot of risk ratio (with 95%-CI) of EOGBS infection in universal strategies versus risk-based strategies.

Any strategy (i.e. risk-based, universal or ‘other’) was associated with a reduced risk of EOGBS infection compared to no strategy (n = 34 studies, RR 0.46 (0.36–0.60), I^2^ = 93%, Chi^2^-test p < 0.001) (Fig. 2 and Supplementary Files 4, 5 and 6). Similarly, risk-based (n = 11 studies, RR 0.65 (0.48–0.87)) and universal strategies (n = 16 studies, RR 0.37 (0.25–0.55)) were also associated with reduced risk of EOGBS infection compared to no strategy (Fig. 3, Fig. 4 and Supplementary Files 4, 5 and 6). However, there was significant evidence for publication bias in the comparison of risk-based vs. no strategy (Supplementary File 6). In direct comparison, universal strategies were significantly associated with a reduced risk of EOGBS infection compared to risk-based strategies (n = 17 studies, RR 0.41 (0.30–0.55), Fig. 5 and Supplementary Files 4, 5 and 6). Results were similar for studies reporting on term incidence and after sensitivity and leave-one-out analyses (Supplementary Files 3, 4, 5 and 6).

Meta-regression demonstrated that there was a correlation between the baseline EOGBS infection incidence and the differences between any strategy and no strategy (β = −0.463, p < 0.001) and between universal and no strategy (β = −0.746, p < 0.001). This indicates that the effect of any or universal strategies compared to no strategy is increased if the incidence in the population is higher. This was not the case in the comparison between risk-based and no strategy (β = −0.328, p = 0.082).

#### Timing of GBS determination

Vergani et al.^i^ reported no significant differences in EOGBS infection between early and late antepartum determination (RR 1.24 (0.25–6.12)), while El Helali et al. reported that intrapartum determination significantly reduced EOGBS infection compared to antepartum determination (RR 0.21 (0.07–0.64)) (Supplementary Files 2.1, 4 and 5).^43^^,^^92^ Meta-regression demonstrated that there was no correlation between timing of GBS determination and the differences between universal and no or risk-based strategies.

#### Additional early-onset infection

Any strategy was associated with a reduced risk of non-GBS EOS compared to no strategy, but individual strategies did not significantly impact non-GBS EOS (Supplementary Files 4, 5 and 6). Similar to EOGBS infection comparisons, any, risk-based and universal strategies were all significantly associated with a reduced risk of all EOS compared to no strategy (Supplementary Files 4, 5 and 6). Universal strategies were associated with a reduced risk of all EOS compared to risk-based strategies (Supplementary Files 4, 5 and 6).

#### EOGBS-related mortality

The pooled EOGBS-related mortality rate during periods with no strategy (0.089 (0.047–0.17), n = 15 studies, I^2^ = 86%) was reduced by more than half after implementation of any strategy (0.028 (0.022–0.036, n = 19 studies, I^2^ = 0%) (Supplementary File 2.3). EOGBS-related mortality rate was similar in periods with risk-based (0.026 (0.019–0.037), n = 6 studies, I^2^ = 36%) and universal strategies (0.028 (0.015–0.054, n = 10 studies, I^2^ = 0%) (Supplementary File 2.3). Likewise, pooled EOGBS case-fatality rate was higher during periods with no strategy (10 (7–15) %, n = 17 studies, I^2^ = 71%) compared to periods with any strategy (6 (5–8) %, n = 20 studies, I^2^ = 7%) and similar in periods with risk-based (5 (4–7) %, n = 8 studies, I^2^ = 0%) and universal strategies (4 (3–6) %, n = 11 studies, I^2^ = 0%) (Supplementary File 2.4).

#### IAP administration

Pooled IAP administration rate during periods with no strategy (8 (3–17) %, n = 3 studies, I^2^ = 100%) more than doubled with any strategy (19 (16–22) %, n = 16 studies, I^2^ = 100%). In the comparison of risk-based (16 (12–20) %, n = 11 studies, I^2^ = 97%) and universal strategies (21 (18–24) %, n = 12 studies, I^2^ = 98%) no significant difference in the rate of IAP administration was found (n = 9 studies, RR 1.29 (0.95–1.75), I^2^ = 99%, Chi^2^-test p < 0.001) (Supplementary File 2.5, 4 and 6).

#### Antimicrobial resistance

In the 11 studies reporting on antimicrobial resistance of EOGBS isolates, there was no resistance to ampicillin, penicillin or other β-lactams but varying resistance to antimicrobials administered in the presence of a penicillin allergy, such as erythromycin and clindamycin, but not vancomycin (Supplementary File 2.6).

## Discussion

Our study adds to the data from previous reviews by expanding the number of included studies and thus the population size, as well as by exploring outcomes not previously reported (non-GBS early-onset sepsis (EOS), mortality and maternal peripartum infection).^12^^,^^13^ In this systematic review and meta-analysis of 72 studies that included more than 10 million live births and pregnant women, all strategies (i.e. any, risk-based, universal and ‘other’ strategies) were associated with a lower risk of early-onset Group B Streptococcal (EOGBS) infection. Universal strategies were associated with a lower risk of EOGBS infection compared to risk-based strategies, while intrapartum antibiotic prophylaxis (IAP) rate was not significantly different between strategies without report of antimicrobial resistance of EOGBS isolates to penicillin or ampicillin. Pooled EOGBS-related mortality was halved in periods with any strategy compared to periods with no strategy and non-GBS EOS incidence decreased after implementation of any strategy. This systematic review and meta-analysis confirms the findings of two previous reviews that universal strategies are more effective in preventing EOGBS infection compared to having no strategy or risk-based strategies.^12^^,^^13^

In this review, evidence was mostly derived from observational studies conducted in high- and high-middle-income countries with historical controls (pre-post implementation studies). These study limitations are reflected in serious risks of bias, often due to potential confounding and potential time bias^j^. Regardless, after sensitivity analyses, in which the data was graded as moderate-level certainty (though limited in the amount of studies included and interpreted with necessary caution), and after leave-one-out analyses, results were similar with respect to EOGBS infection incidence. Other limitations included heterogeneity of the strategies used, which led to classifying all that did not fit the terms ‘risk-based’ or ‘universal’ into an ‘other’ category. This category consisted of at least 10 different strategies, which could not be pooled.

The results of this systematic review and meta-analysis need to be seen in context, by including consideration with regard to cost, feasibility and providers' and women's views. Universal strategies appear to be optimal in preventing EOGBS, with most protocols determining GBS colonisation between 35 and 37 weeks' gestation.^4^^,^^7^^,^^107^ However, inherently, these protocols do not take into account preterm birth occurring before determination, in which EOGBS morbidity and mortality are higher.^4^ It is known that GBS diagnostics with regard to GBS colonisation lose accuracy as time progresses between diagnosis and birth, indicative of possible transient colonisation.^108^ Earlier screening would, therefore, signify a less accurate prediction of EOGBS infection risk for infants, but our findings do not support this theory. Although intrapartum determination might lower EOGBS infection compared to antenatal determination, intrapartum testing is not yet widely accessible and results are not always readily available to provide IAP 4 h prior to birth; hence, more research to understand the potential harms and benefits is needed.

Discussion concerning the potential for IAP strategies to contribute to harm, such as increased non-GBS neonatal sepsis have featured prominently in this space, in addition to possible overtreatment and rising antimicrobial resistance with universal strategies, on account of associations between early antibiotic exposure and altered gut microbiome, asthma and obesity.^14^, ^15^, ^16^^,^^109^^,^^110^ Interestingly, instead of an increase in non-GBS EOS, we observed that implementation of any strategy was associated with a lower risk of non-GBS EOS. The studies reporting an increasing incidence of gram-negative sepsis (particularly *E. coli*) primarily focused on very low-birthweight infants, while studies in premature and term infants (not included in our meta-analysis) did not observe such an increase, similar to our findings.^16^^,^^30^^,^^109^^,^^111^ Our findings also suggest that antimicrobial resistance in EOGBS isolates is confined to antimicrobials administered in the presence of a penicillin allergy. Hence, penicillin and ampicillin might still be the best choice for IAP in preventing vertical transmission of GBS, especially since recent evidence suggests that clindamycin is less effective than ampicillin against vertical transmission of GBS.^112^ Moreover, our findings provide some reassurance that universal strategies are not likely to result in significantly higher antibiotic exposure compared to risk-based strategies.

Despite evidence for this systematic review and meta-analysis predominantly deriving from studies conducted in high and high-middle-income countries, GBS prevention strategies might also lower the high EOGBS burden in low- and lower-middle income countries. Low and lower-middle-income countries more commonly have no strategy for IAP, partly due to lack of resources and inadequate infrastructure for diagnostic screening and testing, while they have higher EOGBS morbidity and mortality.^113^, ^114^, ^115^ In our review, we included one study that reported on the adoption of a strategy in a lower-middle-income country^k^ (India).^83^ After the adoption of a risk-based strategy, EOGBS infection incidence significantly decreased, particularly in premature infants, without any antimicrobial resistance for penicillin and ampicillin in EOGBS isolates.^83^ As these results are similar to the findings in this review, IAP strategies might prove beneficial in these settings, though more research in low- and lower-middle income countries is necessary.

Worldwide annual EOGBS burden is about 205,000 affected newborn infants, of which a significant proportion results in death.^115^ Any IAP strategy aimed to lower this EOGBS burden could reduce risk of EOGBS infection and non-GBS sepsis, while also lowering EOGBS-related mortality. Specifically, universal strategies likely lead to a larger reduction in EOGBS infection compared to risk-based strategies while a similar proportion of pregnant women receive IAP. Considering that EOGBS isolates were not resistant to ampicillin or penicillin, altogether, our findings do not support evidence that IAP strategies, and in particular, universal strategies, are associated with explicit harm, though data was derived from observational studies.

Currently, the randomised multicentre GBS3 (ISRCTN49639731) trial is being conducted in the United Kingdom comparing risk-based and universal strategies (antepartum and intrapartum). It is the first trial to concurrently investigate (cost)-effectiveness of the different strategies and will provide valuable insight into the optimal GBS prevention strategy.

All in all, EOGBS infection is still one of the important issues in perinatal health. This systematic review and meta-analysis has demonstrated that, according to the most up-to-date evidence yet, universal strategies are likely the most optimal choice to reduce the worldwide EOGBS burden.

## Contributors

Conceptualisation: TJRP, GFH, CA, TL, ABtP, VB and TvdA. Data curation: TJRP. Formal analysis: TJRP. Investigation: TJRP, GFH, YC, CA, TL and VB. Methodology: TJRP, GFH, CA, TL, ABtP, VB and TvdA. Project administration: TJRP, TL, TvdA. Resources and software: Not relevant for this review. Supervision: TL and TvdA. Validation: GFH. Visualisation: TJRP. Writing—original draft: TJRP and GFH. Writing—review & editing: YC, CA, TL, ABtP, VB and TvdA. All authors reviewed and approved the final version before submission. All authors had access to all data used in this study, approved the final version of the manuscript, and accepted the responsibility for the decision to submit the manuscript for publication.

## Data sharing statement

All data were presented in the manuscript and supplementary files.

## Declaration of interests

Authors have no competing or conflicts of interests to declare.

## Footnotes

h If data was already published in other articles, then the study with the largest sample size was chosen and included.

## Footnotes

i Vergani et al. used a combination strategy consisting of IAP administration to pregnant women with GBS colonisation and to those with risk-factors.

j The time bias could consist of possible improvements in care over time, but is mitigated by increased viability of premature infants and subsequent survival that could increase the amount of EOGBS cases over time. Therefore, it is unclear whether this bias favours the effect of the intervention or not.

k This study was not included in the synthesis of EOGBS infection, because no raw data was available. The study characteristics, results and risk of bias are mentioned in the corresponding tables.
