# Supplementary material for: Intrapartum antibiotic prophylaxis to prevent Group B streptococcal infections in newborn infants: a systematic review and meta-analysis comparing various strategies
Source: eClinicalMedicine. 2024 Jul 28;74:102748. doi: 10.1016/j.eclinm.2024.102748 (PMC11577566; doi:10.1016/j.eclinm.2024.102748)

# Forest plots for meta-analysis comparisons

Term EOGBS:

Forest plot, term EOGBS, any vs no strategy


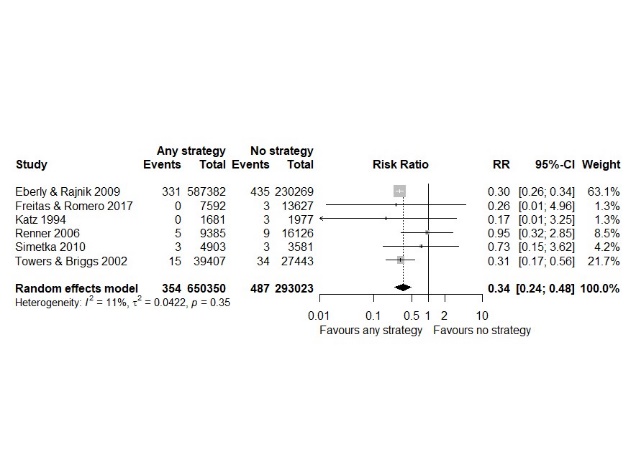


Forest plot, term EOGBS, risk vs no strategy


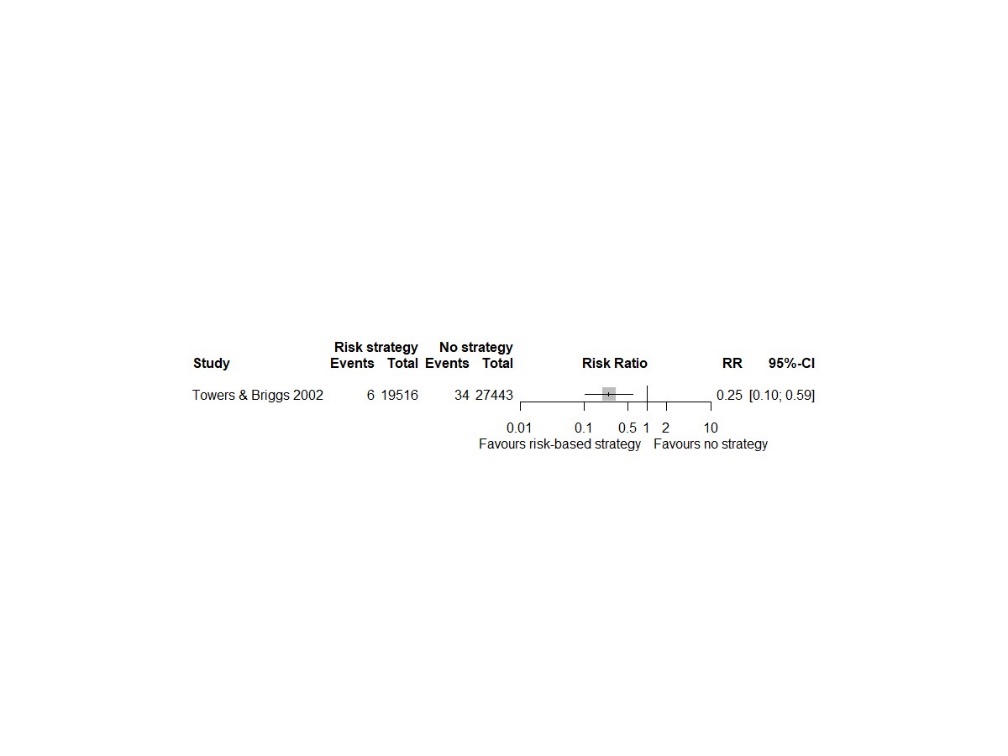


Forest plot, term EOGBS, universal vs no strategy


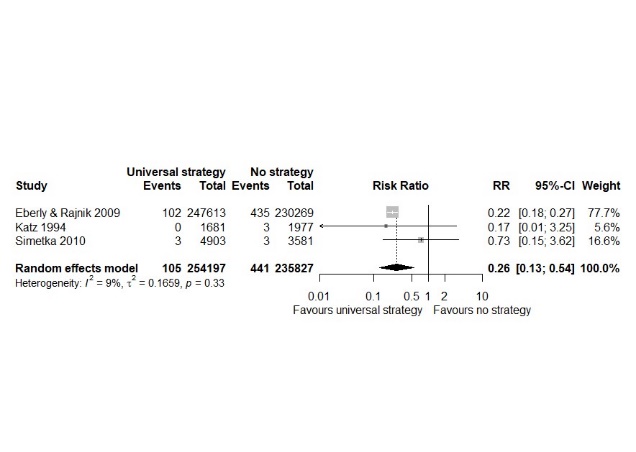


Forest plot, term EOGBS, universal vs risk strategies


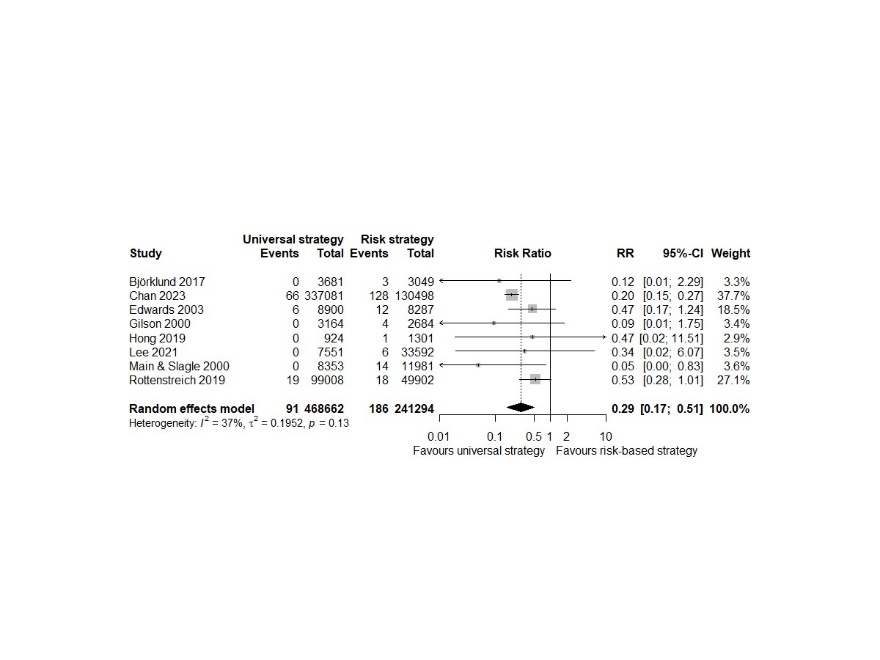


Sensitivity analysis (SA):

Forest plot, SA EOGBS, any vs no strategy


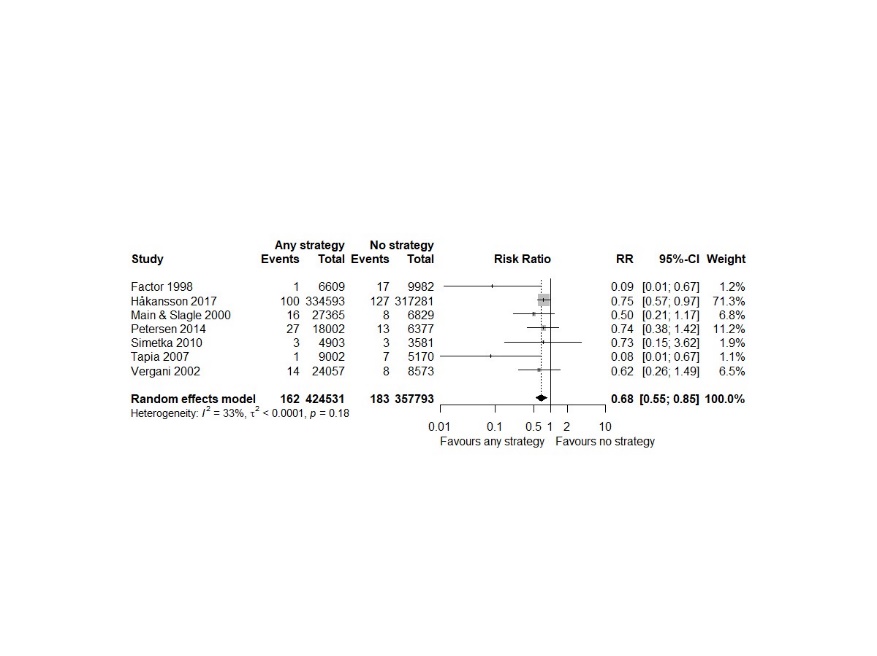


Forest plot, SA EOGBS, risk vs no strategy


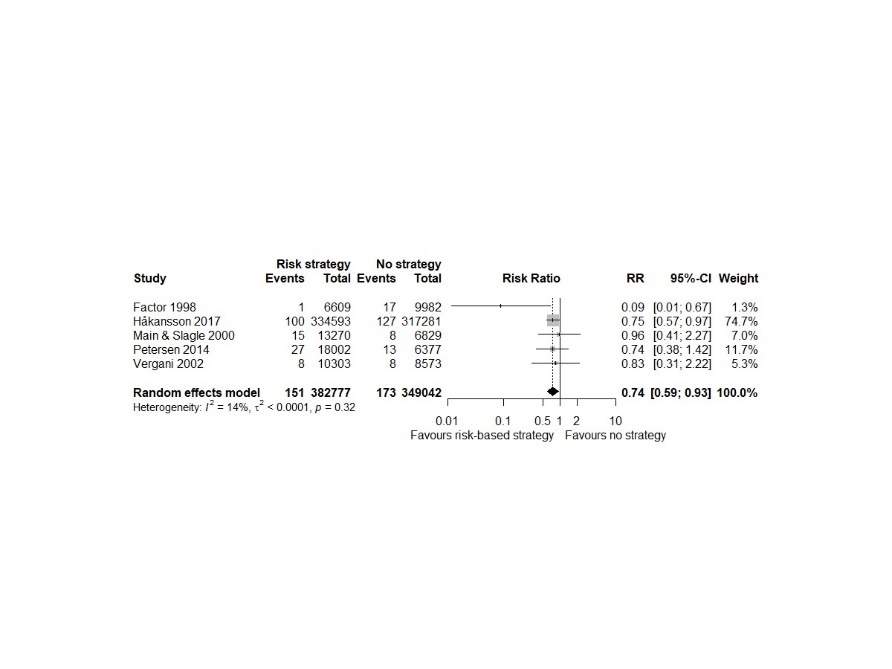


Forest plot, SA EOGBS, universal vs no strategy


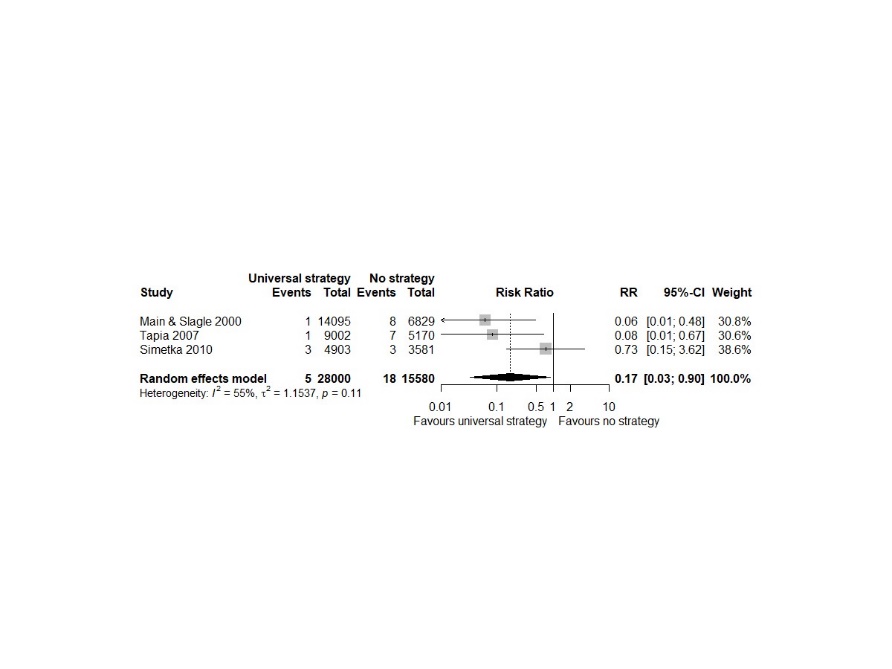


Forest plot, SA EOGBS, universal vs risk strategies


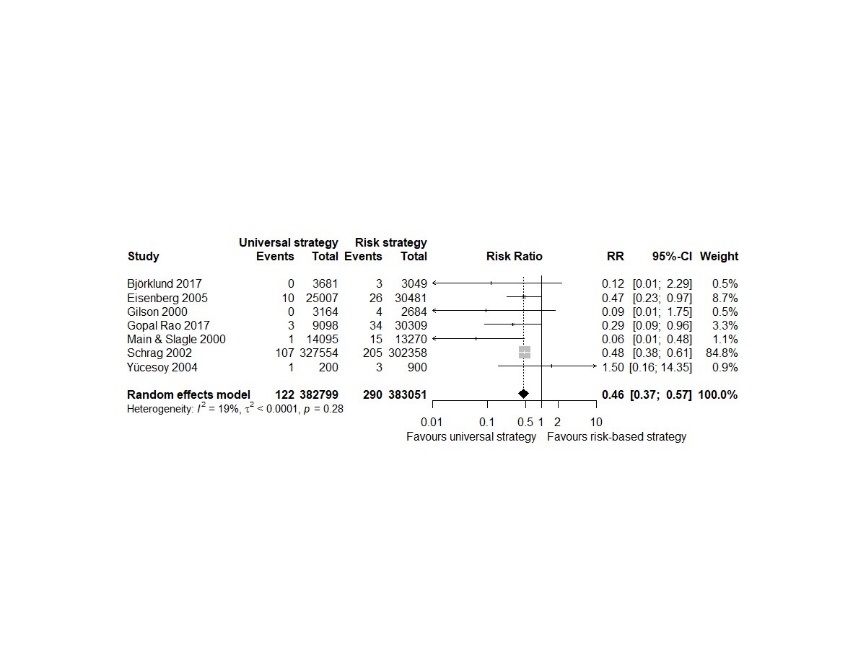


Leave-one-out analysis (L1OA):

Forest plot, L1OA EOGBS, any vs no strategy


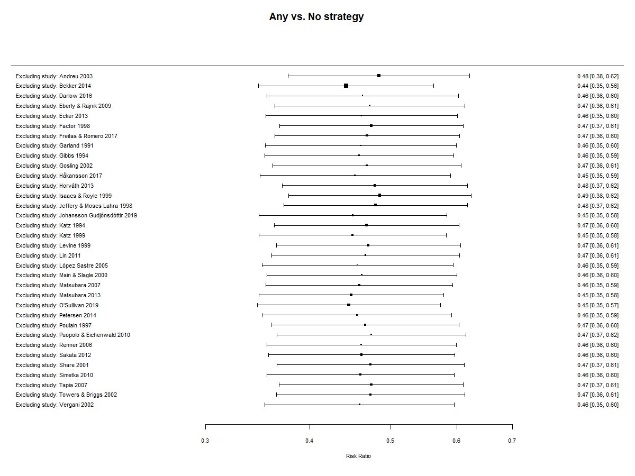


Forest plot, L1OA EOGBS, risk vs no strategy


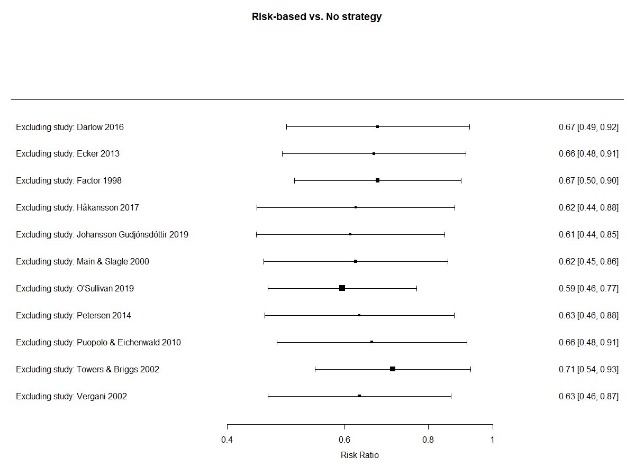


Forest plot, L1OA EOGBS, universal vs no strategy


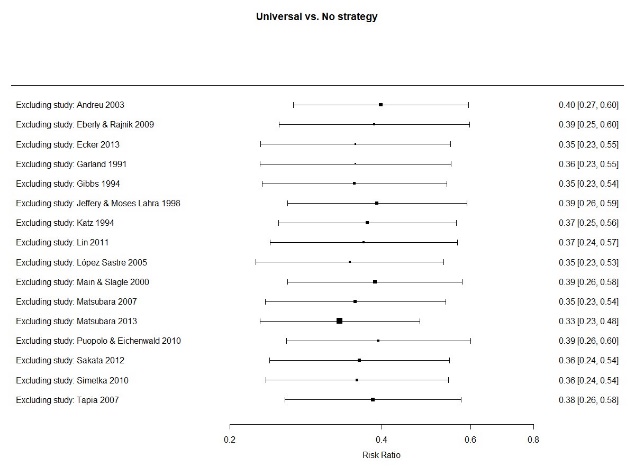


Forest plot, L1OA EOGBS, universal vs risk strategies


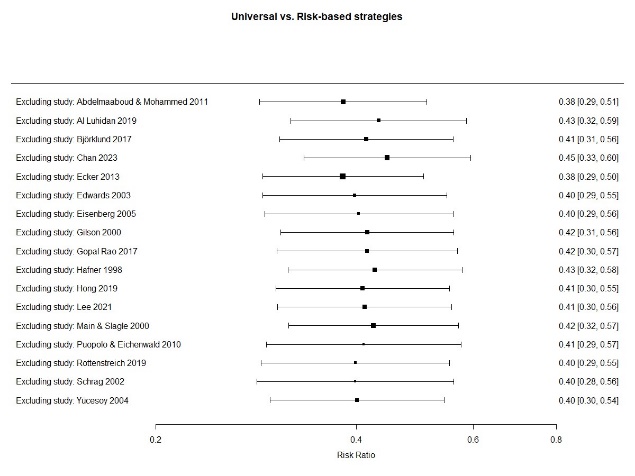


Timing of determination:

Forest plot, EOGBS, early vs late antenatal determination


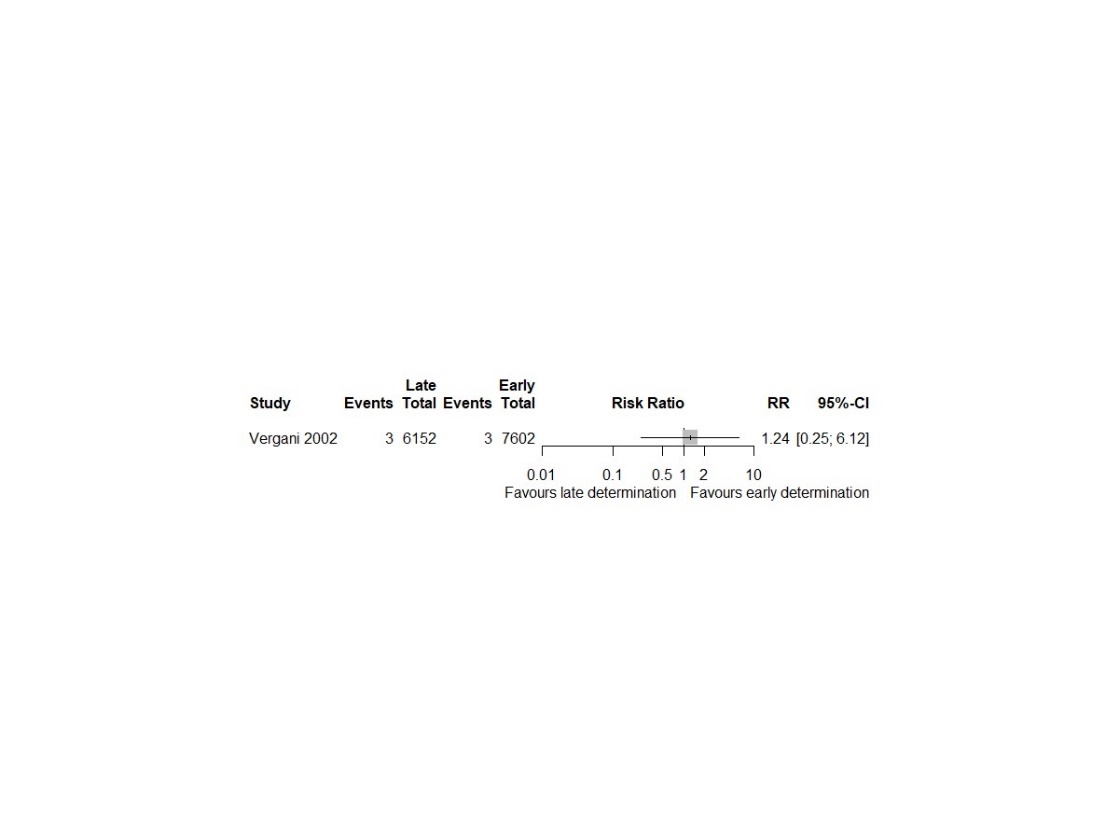


Forest plot, EOGBS, intrapartum vs antepartum determination


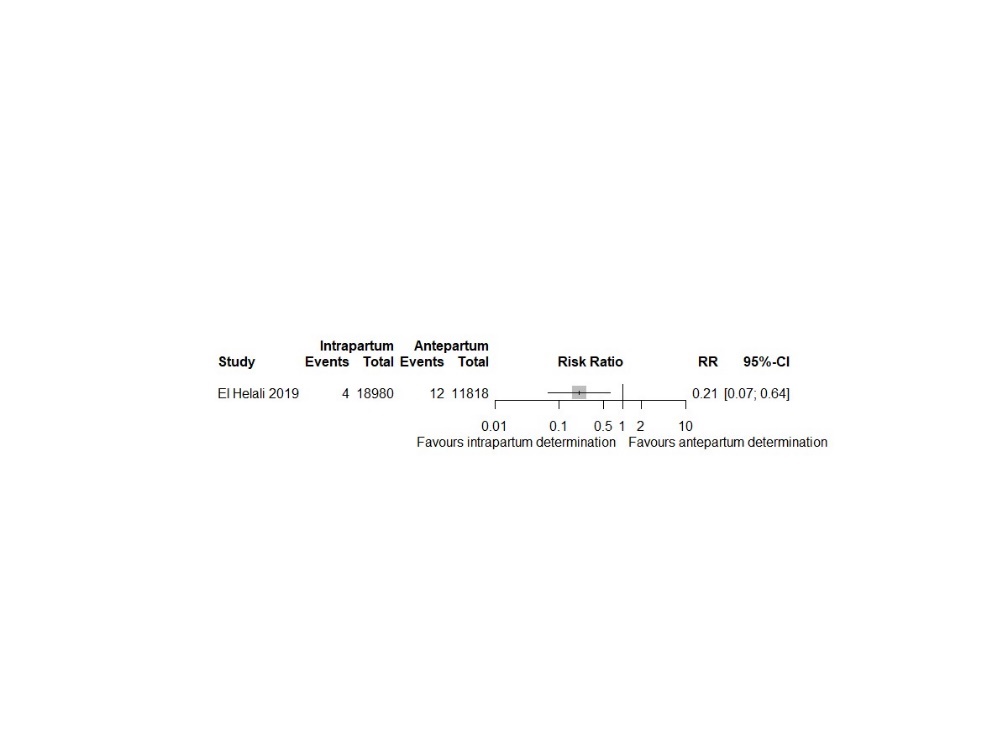


Non-GBS EOS:

Forest plot, Non-GBS EOS, any vs no strategy


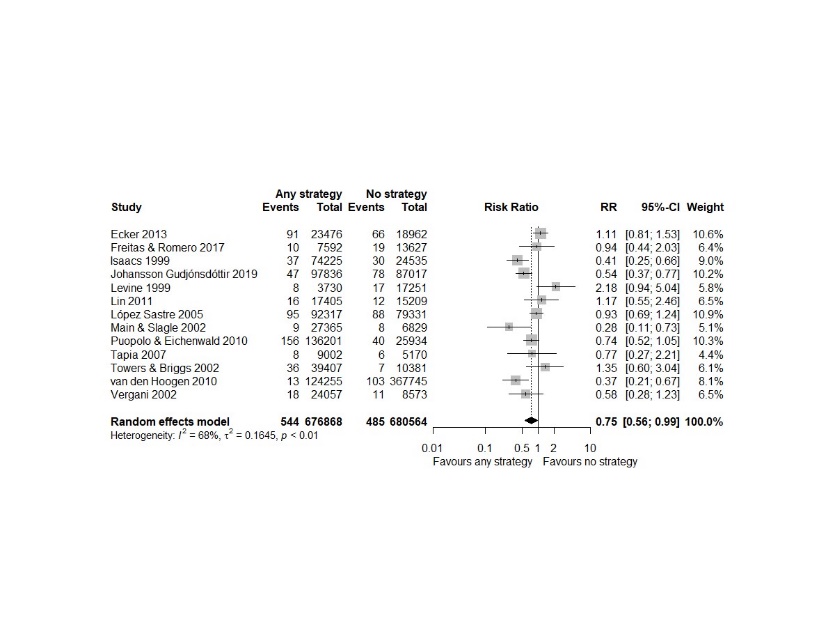


Forest plot, Non-GBS EOS, risk vs no strategy


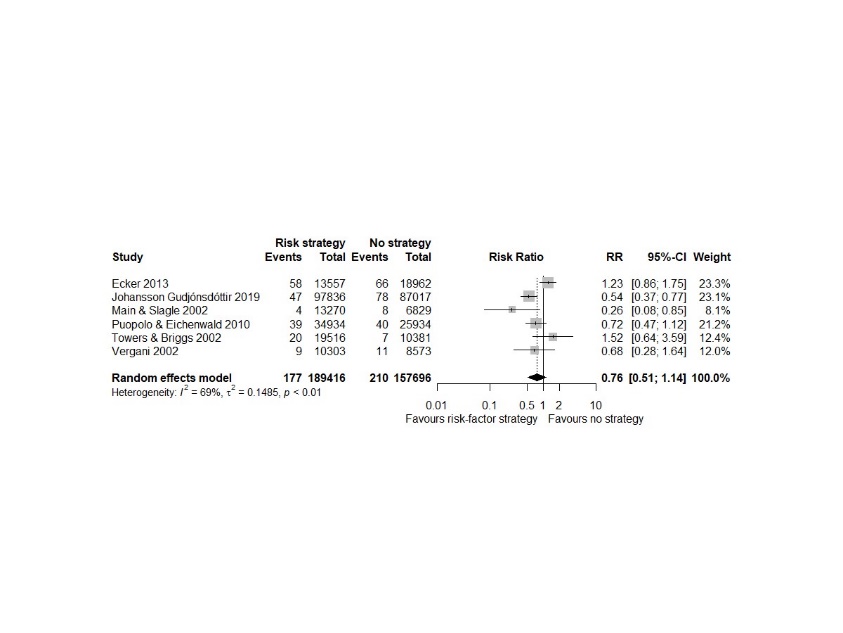


Forest plot, Non-GBS EOS, universal vs no strategy


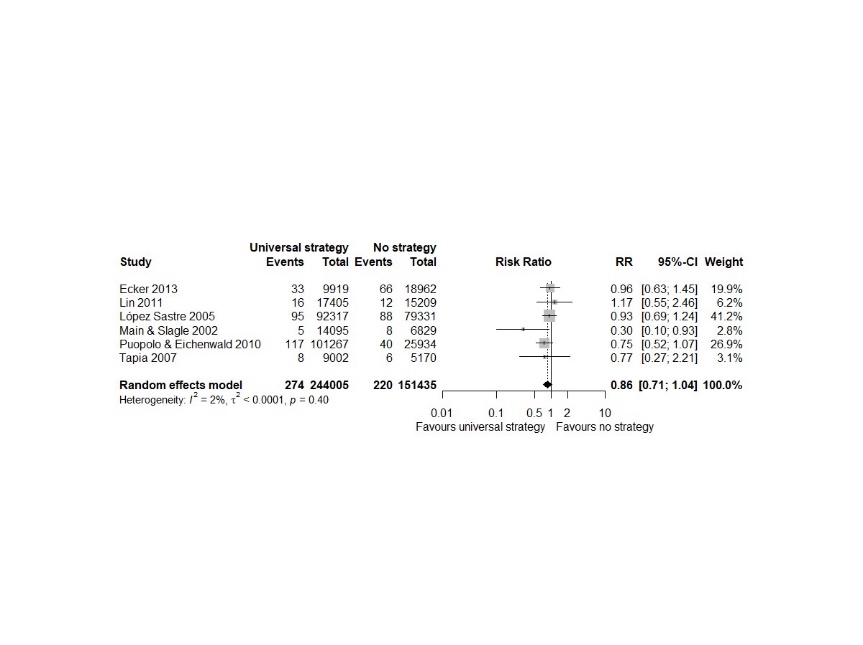


Forest plot, Non-GBS EOS, universal vs risk strategies


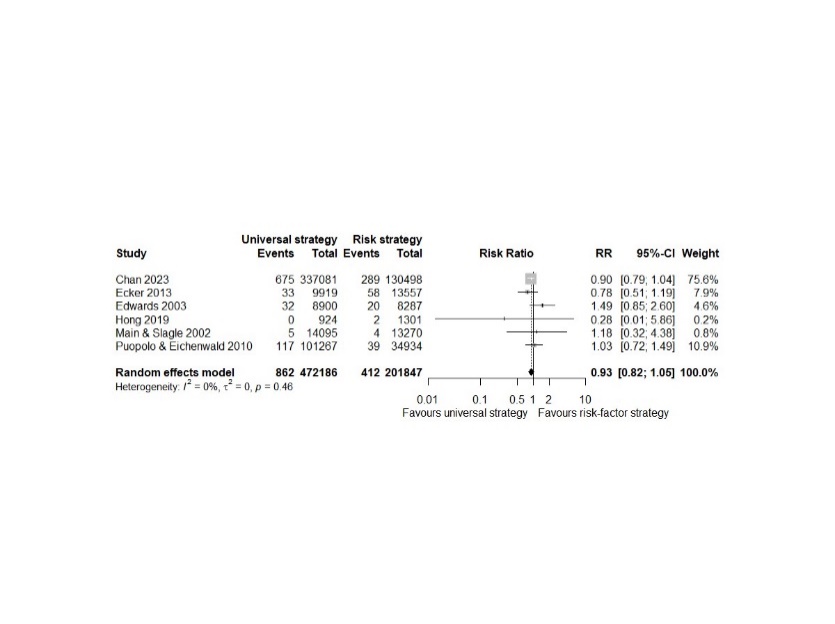


All EOS:

Forest plot, all EOS, any vs no strategy


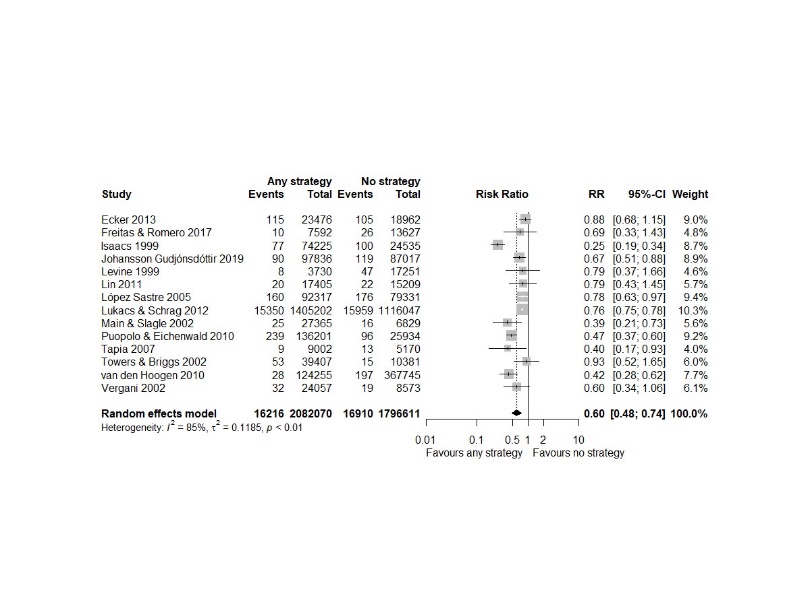


Forest plot, all EOS, risk vs no strategy


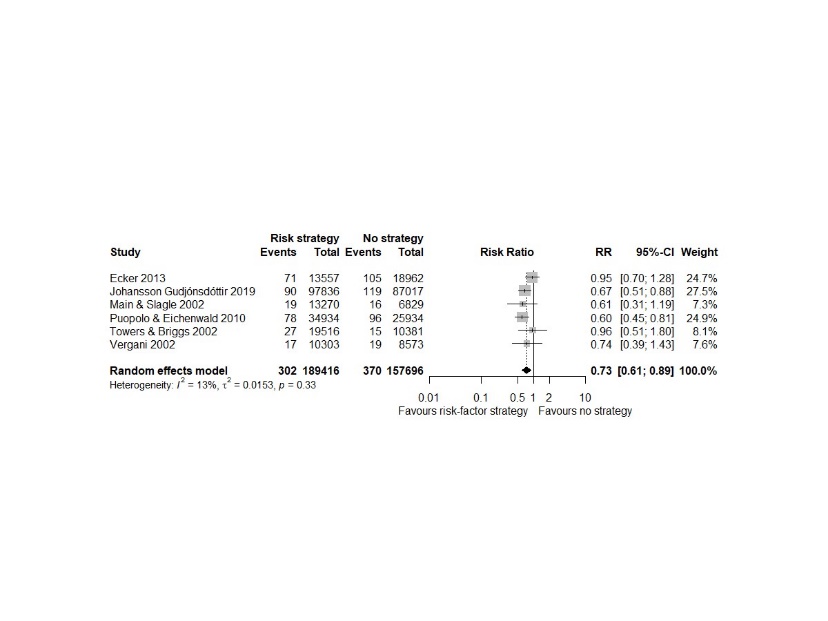


Forest plot, all EOS, universal vs no strategy


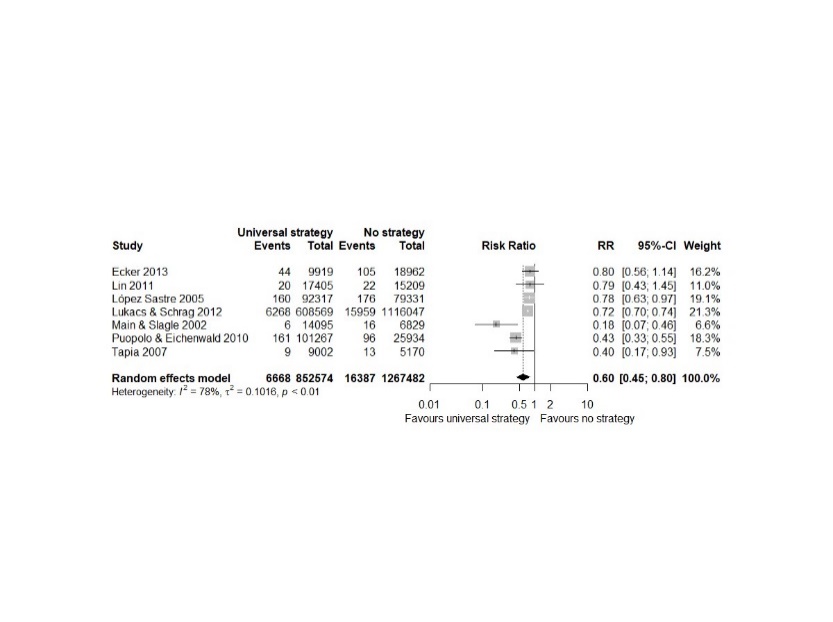


Forest plot, all EOS, universal vs risk strategies


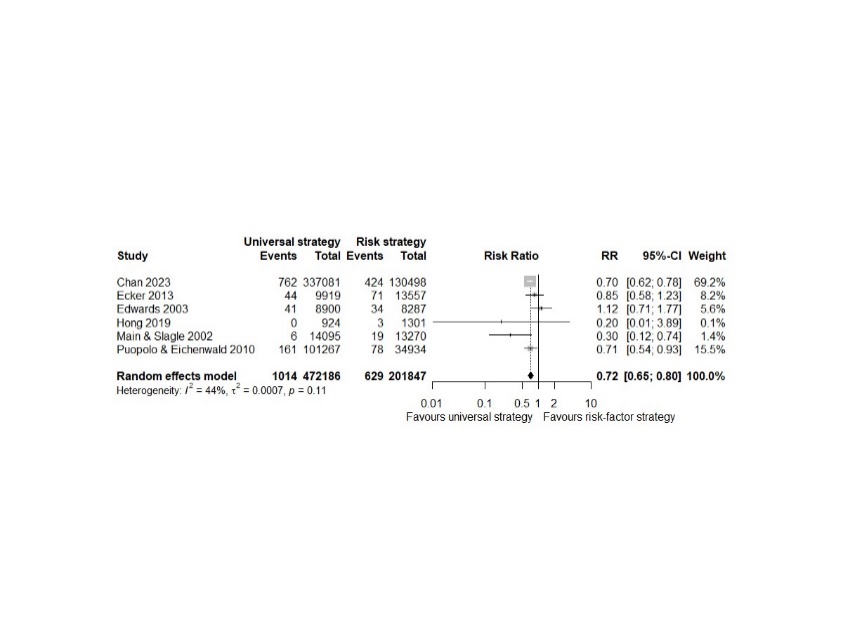


IAP:

Forest plot, IAP, universal vs risk strategies


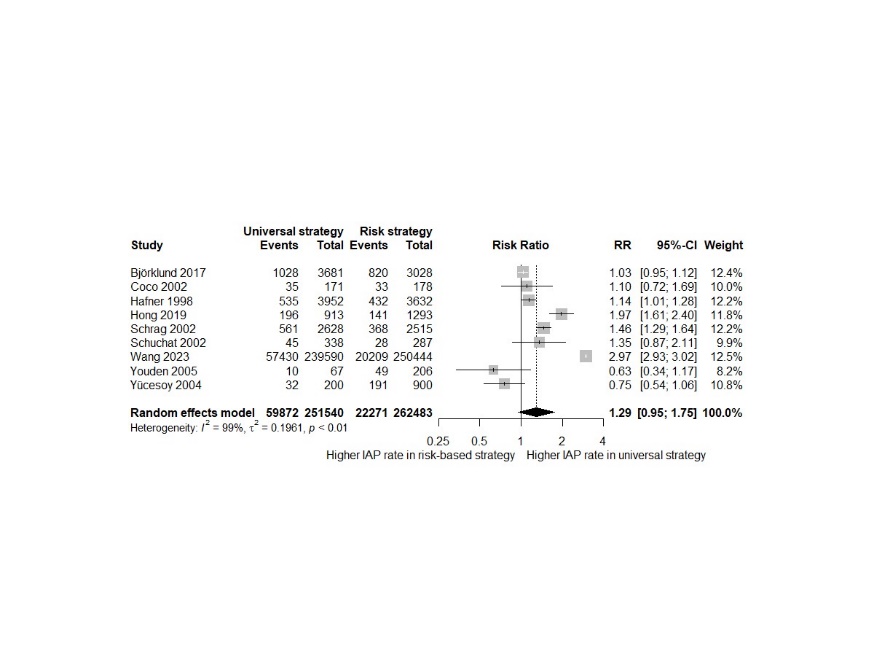

Supplement: Supplementary File 4 [file mmc4.docx]
