# Supplementary material for: Intrapartum antibiotic prophylaxis to prevent Group B streptococcal infections in newborn infants: a systematic review and meta-analysis comparing various strategies
Source: eClinicalMedicine. 2024 Jul 28;74:102748. doi: 10.1016/j.eclinm.2024.102748 (PMC11577566; doi:10.1016/j.eclinm.2024.102748)

# Publication bias funnel plots for meta-analysis comparisons

Publication bias funnel plot, EOGBS, any vs no strategy


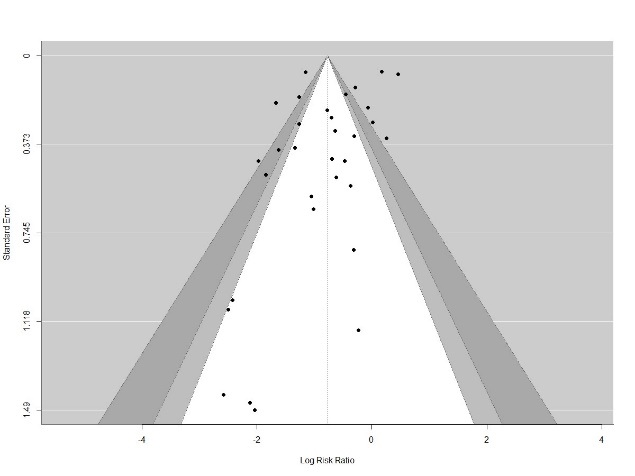


Publication bias funnel plot, EOGBS, risk vs no strategy


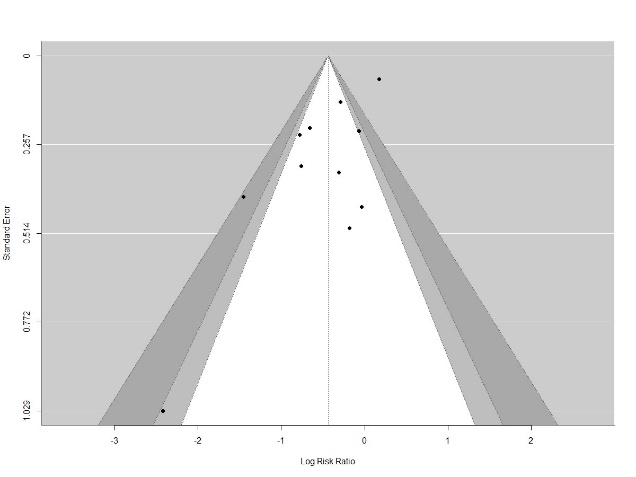


Publication bias funnel plot, EOGBS, universal vs no strategy


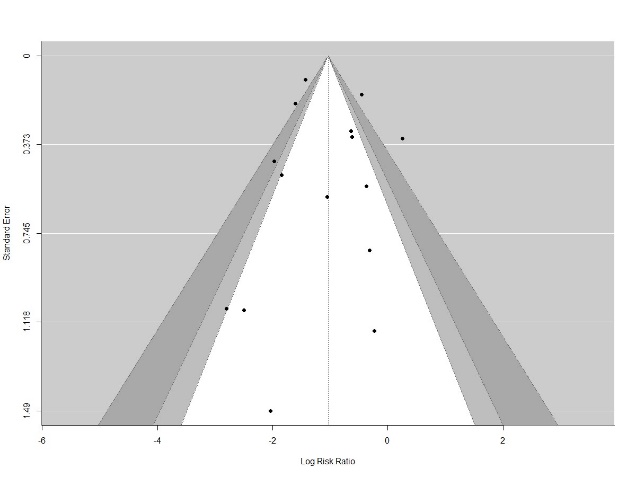


Publication bias funnel plot, EOGBS, universal vs risk strategies


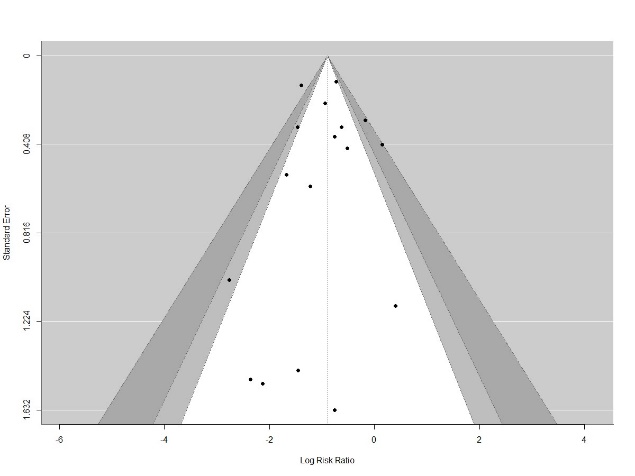


Publication bias funnel plot, Non-GBS EOS, any vs no strategy


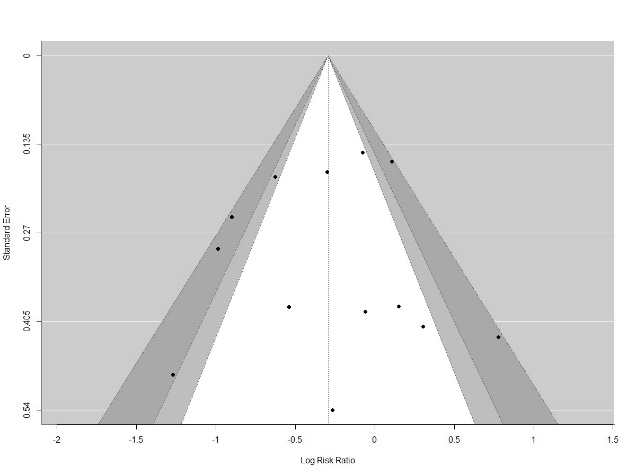


Publication bias funnel plot, All EOS, any vs no strategy


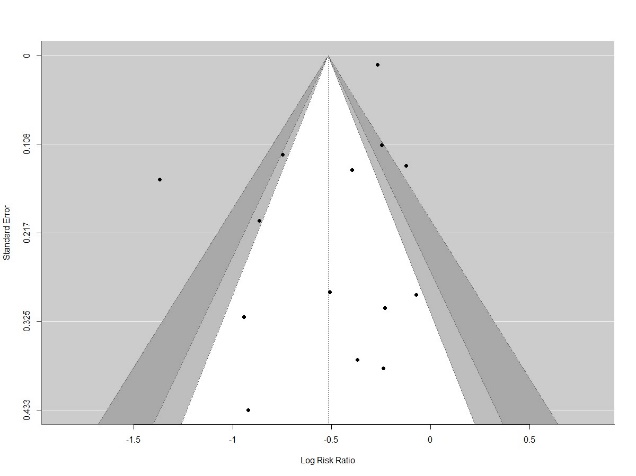

Supplement: Supplementary File 5 [file mmc5.docx]
